# Therapeutic Consequences of ^68^Ga-PSMA-11-PET/CT in Prostate Cancer in Correlation to the Gleason Score, PSA Value, and D’Amico-Defined Risk Groups

**DOI:** 10.3390/cancers17121944

**Published:** 2025-06-11

**Authors:** Friederike Eilsberger, Ali Ebrahimifard, Florian Torsten Spiegel, Behrooz Hooshyar Yousefi, Shamim Bagheri, Wadim Bowl, Qi Wang, Andreas Pfestroff, Laura Müller, Markus Luster, Pietro Di Fazio, Damiano Librizzi

**Affiliations:** 1 Department of Nuclear Medicine, University Hospital Marburg, Philipps University Marburg, 35043 Marburg, Germany; ebrahima@staff.uni-marburg.de (A.E.); florian.spiegel@klinikum-fulda.de (F.T.S.); b.h.yousefi@uni-marburg.de (B.H.Y.); shamim.bagheri@uni-marburg.de (S.B.); wadim.bowl@radiol.med.uni-giessen.de (W.B.); wangqi2@students.uni-marburg.de (Q.W.); pfestrof@med.uni-marburg.de (A.P.); luster@med.uni-marburg.de (M.L.); difazio@med.uni-marburg.de (P.D.F.); 2 Department V, Hospital Fulda, 36043 Fulda, Germany; 3 Department of Urology, University Hospital Marburg, Philipps University Marburg, 35037 Marburg, Germany; laura.mueller2@uk-gm.de; 4 Department of Nuclear Medicine, Protestant Hospital of Bethel Foundation, 33611 Bielefeld, Germany; librizzi@gmx.de

**Keywords:** PSMA, PET/CT, Gleason score, PSA, D’Amico, risk group, prostate cancer

## Abstract

**Background/Objectives**: The aim of this study was to demonstrate the importance of ^68^Gallium (^68^Ga)–prostate specific membrane antigen (PSMA)–positron emissions tomography (PET)/computed tomography (CT)(PET/CT) in prostate cancer patients for therapy management with individual analyses regarding the Gleason score, prostate specific antigen (PSA) value, and the risk groups defined by D’Amico. **Methods**: We retrospectively analyzed 562 ^68^Ga-PSMA-11-PET/CT examinations performed from January 2015 to March 2023 at University Hospital Marburg. We assessed treatment changes post ^68^Ga-PSMA-11-PET/CT and categorized the cases based on PSA values, Gleason scores, and D’Amico risk groups. **Results**: In 415/562 (73.8%) of ^68^Ga-PSMA-11-PET/CT examinations, a modification in the therapy concept was recorded. Patients categorized as high risk or patients with Gleason scores of 7 through 10 or with PSA levels above 0.5 ng/mL (particularly within the ranges of 1.01–2 ng/mL, 3.01–5 ng/mL, and values exceeding 10 ng/mL) demonstrated a statistically significant association with treatment change. While no evidence of the disease was found most frequently in 38% of cases in the “Therapy continued without explicit reference” group, in the group with the adapted therapy, there was a considerable higher proportion of local tumors (19.2%) compared to the other groups (4.4% and 1.4%). **Conclusions**: Our results show the high impact of ^68^Ga-PSMA-PET/CT for patients with prostate cancer regarding therapy management planning, which is even more important for some patient groups.

## Simple Summary

In prostate cancer, specific imaging using positron emission tomography (PET)/computed tomography (CT)(PET/CT) with the prostate-specific membrane antigen (PSMA) can be used to detect the extent of the tumor and metastases. Our work examines the effects of this diagnostic tool on therapy with regard to the Gleason score, the PSA value, and the risk groups. We determined a change in therapy in 73.8% of the ^68^Ga-PSMA-PET/CT examinations. Patients with a high risk or higher Gleason score or with PSA levels above 0.5 ng/mL demonstrated a statistically significant association with treatment change. The absence of disease was most commonly observed in 38% of cases within the “Therapy continued without explicit reference” group, and the “Therapy modified” group showed a higher incidence of local tumors (19.2%) compared to the other groups. Our findings highlight the high influence of ^68^Ga-PSMA-PET/CT on therapy management planning for patients with prostate cancer.

## 1. Introduction

In recent years, ^68^Gallium (^68^Ga)-prostate-specific membrane antigen (PSMA) positron emissions tomography (PET)/computed tomography (CT) has become firmly established in the clinical routine of prostate cancer diagnostics. Several studies have shown the excellent sensitivity and specificity of this diagnostic method, especially in patients with biochemical recurrence [1]. ^68^Ga-PSMA-PET/CT has also been included in the 2024 German prostate carcinoma guideline for recurrence and primary diagnosis in high-risk patients [2]. An adequate individual therapy decision should be built based on the most accurate diagnostics available. The recent literature shows a wide range of the importance of ^68^Ga-PSMA-PET/CT for further therapy planning. A recent meta-analysis by Han et al., including 15 studies and 1163 patients, revealed a pooled proportion of management changes of 54% (95% confidence interval 47–60%) [3]. However, real-world experience from the interdisciplinary tumor boards suggests that ^68^Ga-PSMA-PET/CT is in the majority of cases decisive for the further course of treatment. Our study aims to show the influence of ^68^Ga-PSMA-11-PET/CT on further therapy planning in clinical practice in a tertiary treatment center, with an analysis for the potential correlations to different Gleason scores, PSA values, and D’Amico-defined risk groups.

## 2. Materials and Methods

### 2.1. Patients

We included 562 ^68^Ga-PSMA-11-PET/CT examinations, which were performed between January 2015 and March 2023 at University Hospital Marburg, from patients who underwent both this imaging and a follow-up in our department or were discussed following the examination by the interdisciplinary tumor board to assess the therapy before and after the examination. Treatment recommendations were developed by the interdisciplinary tumor board in the presence of all the specialist disciplines involved, such as urology, radiation oncology, nuclear medicine, oncology, pathology, and radiology. Prostate-specific antigen (PSA) values were recorded for 482 of the examinations, D’Amico risk groups were determinable in 377 examinations, and Gleason scores were documented for 422 of the examinations.

The basic characteristics of the patients can be found in Table 1.

### 2.2. Investigation Protocol

^68^Ga-PSMA-11-PET/CTs were performed according to the recommendation of the German Society of Nuclear Medicine, using Siemens Biograph 6 True Point. This study was approved by the local ethics committee (AZ.: 24-217RS and 24-217_1-RS). Patients received 188 (106–343) MBq of ^68^Ga-PSMA-11-PET/CT, and the scans were performed 60 (30–145) min after application. As there was no valid European or German guideline at the time of the first ^68^Ga-PSMA-11-PET/CT examinations, patients were given individual activities based on the study situation valid at the time. The EANM/SNMMI guideline published in 2017 also emphasized that the optimal activity was still being debated; the majority of published data are based on an injected activity of approximately 1.8–2.2 MBq per kilogram of body weight, which, considering the applied activities, is achieved [4]. This recommendation can also be found in the currently valid German S1 guideline [5].

### 2.3. Analysis

Initially, all 562 scans were assigned if the examination led to a change in treatment recommendations. In a second step, we re-examined the cases marked with “no change in therapy” to determine whether the continuation of the previous therapy was justified with reference to ^68^Ga-PSMA-11-PET/CT. We categorized the cases with available PSA values (n = 482) into the PSA ranges defined by Afshar-Oromieh et al. [6], the cases with Gleason scores available (n = 422 cases) into Gleason score groups, and classified the patients as far as possible in the risk groups by D’Amico et al. (n = 377 cases) for further analysis. The risk groups implemented by D’Amico were defined as follows: a low risk profile for PSA < 10 ng/mL and Gleason score ≤ 6, a medium risk profile for PSA 10–20 ng/mL or Gleason score = 7, and a high risk profile for PSA > 20 ng/mL or Gleason score > 7 [7].

The variables evaluated included the D’Amico risk classification (low, intermediate, high), Gleason score (ranging from 5 to 10), and serum PSA levels, which were stratified into clinically relevant intervals: ≤0.2, 0.21–0.5, 0.51–1, 1.01–2, 2.01–3, 3.01–5, 5.01–7, 7.01–10, and >10 ng/mL. The primary outcome variable was whether or not the patient underwent a change in treatment during follow-up. To evaluate statistical significance, a one-sample t-test was applied to each subgroup, comparing the observed proportion of patients who experienced a treatment change against a reference proportion. A *p*-value of less than 0.05 was considered statistically significant.

The data were organized using Microsoft Excel (Version 16.78.3, Redmond, WA, USA) and all statistical analyses were conducted using GraphPad Prism version 10.0 (GraphPad Software, San Diego, CA, USA).

## 3. Results

### 3.1. Adjustments in Treatment After the ^68^Ga-PSMA-11-PET/CT

**Figure 1:**
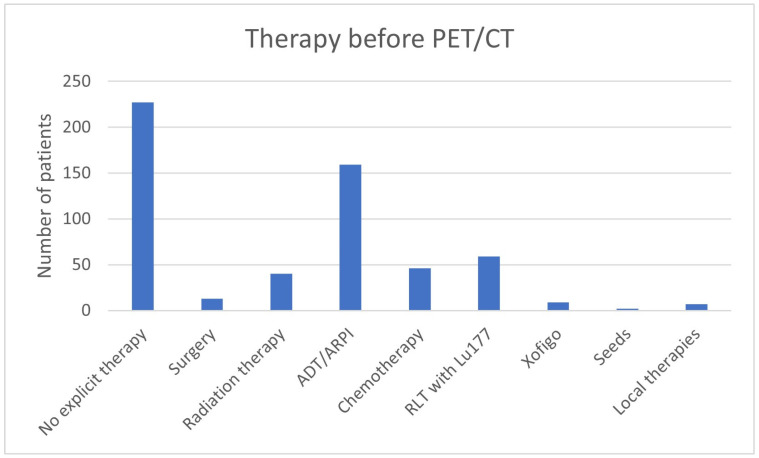
Therapy prior to ^68^Ga-PSMA-11-PET/CT.

**Figure 2:**
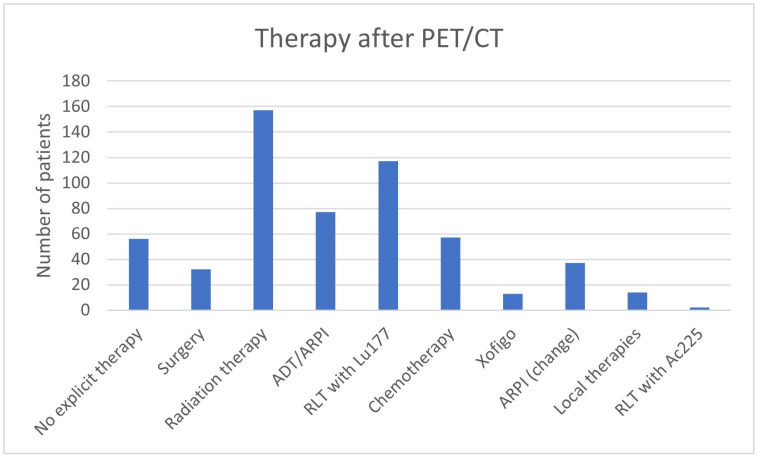
Therapy post ^68^Ga-PSMA-11-PET/CT.

In 415/562 (73.8%) patients, the therapy was adjusted after the ^68^Ga-PSMA-11-PET/CT (Table 2, Figure 1 and Figure 2). No change in therapy was implemented in 147/562 (26.2%) patients. In 68/147 (46.26%), the current treatment was explicitly continued with reference to the ^68^Ga-PSMA-11-PET/CT.

### 3.2. Impact of D’Amico Risk 

**Figure 3:**
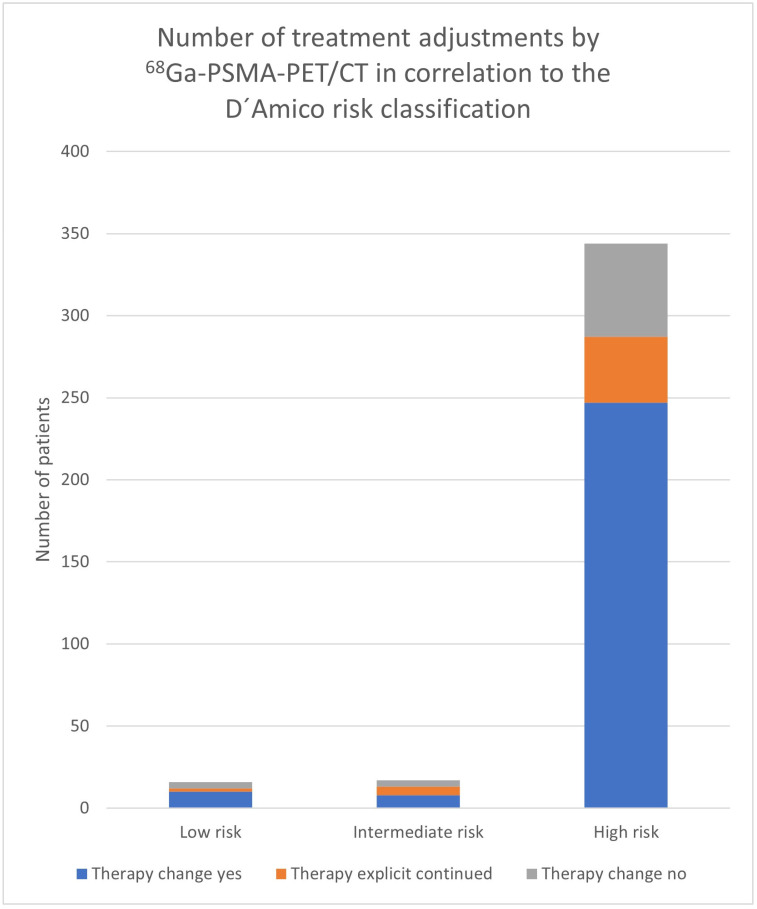
Number of treatment adjustments by ^68^Ga-PSMA-11-PET/CT in correlation to the D’Amico risk classification.

**Figure 4:**
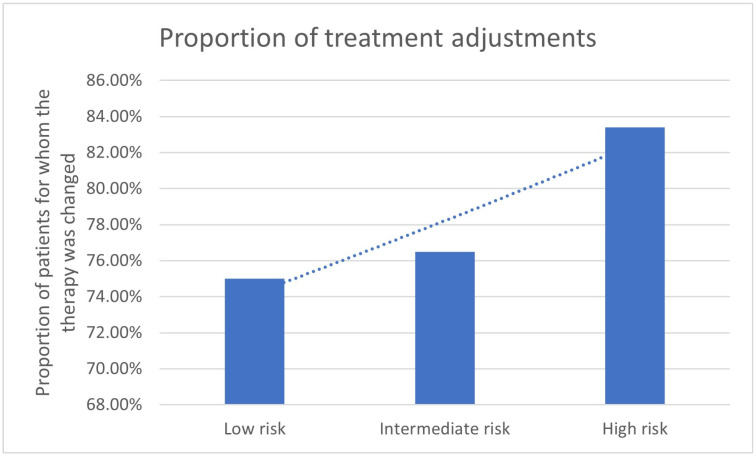
Proportion of treatment adjustments changed by ^68^Ga-PSMA-11-PET/CT in correlation to the D’Amico risk classification.

The analysis of the results of the different risk groups shows differing percentages for the adaptation of therapy management as a result of ^68^Ga-PSMA-11-PET/CT. In patients with low risk, a treatment adaptation was performed in 10/16 (62.5%) of patients, in the intermediate-risk group, in 8/17 (47.1%), and in the high-risk group, in 247/344 (71.8%) of patients (Table 3). The decision was made through the ^68^Ga-PSMA-11-PET/CT in 75% in the low-risk group, in 76.5% in the intermediate-risk group, and in 83,4% in the high-risk group (Table 3, Figure 3 and Figure 4). The number and proportion of treatment adjustments by ^68^Ga-PSMA-11-PET/CT in correlation to the D’Amico risk classification are shown in Figure 3 and Figure 4.

According to the analysis based on the D’Amico risk classification, only patients categorized as high risk demonstrated a statistically significant association with treatment change (*p* < 0.0001), whereas no significant associations were observed in the low- and intermediate-risk groups (*p* = 0.33; *p* = 0.82), although the group sizes here were also considerably smaller.

**Figure 5:**
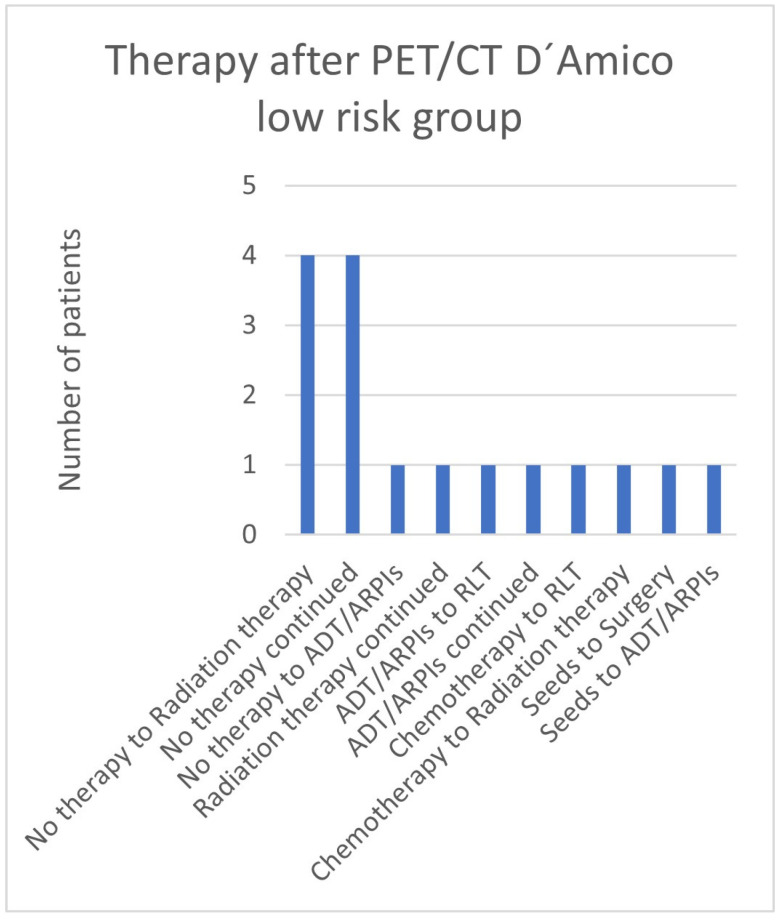
Treatment adjustments by ^68^Ga-PSMA-11-PET/CT in the D’Amico low-risk group.

In the low-risk group, 4/16 (25.0%) patients changed from “no therapy” to radiation therapy or continued “no therapy”. One (6.3%) patient each continued radiation therapy or androgen deprivation therapy/androgen receptor pathway inhibitor (ADT/ARPI) or switched from “no therapy” to ADT/ARPI, from ADT/ARPI to radioligand therapy (RLT), from chemotherapy to RLT or radiation therapy, or from seeds to surgery or ADT/ARPI (Figure 5).

**Figure 6:**
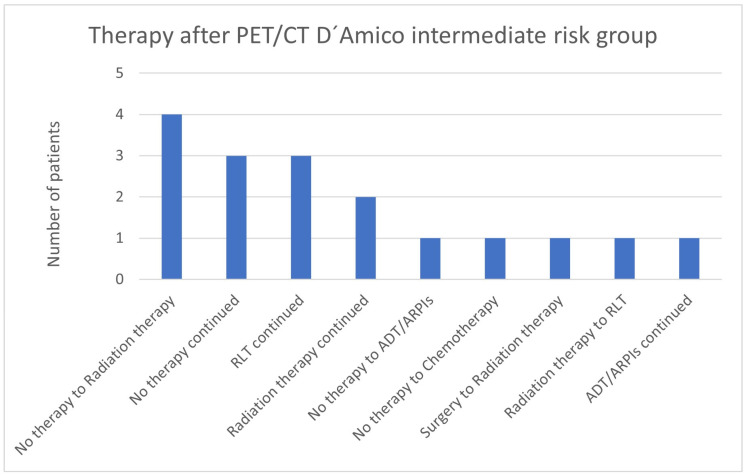
Treatment adjustments by ^68^Ga-PSMA-11-PET/CT in the D’Amico intermediate-risk group.

In total, 4/17 (23.5%) patients in the intermediate-risk group changed from “no therapy” to radiation therapy, 3 (17.6%) continued “no therapy” or RLT, and 2 (11.8%) continued radiation therapy (Figure 6). Respectively, one case (5.9%) was modified from “no therapy” to ADT/ARPI or chemotherapy, from surgery to radiation therapy, and from radiation therapy to RLT, and one continued on ADT/ARPI.

**Figure 7:**
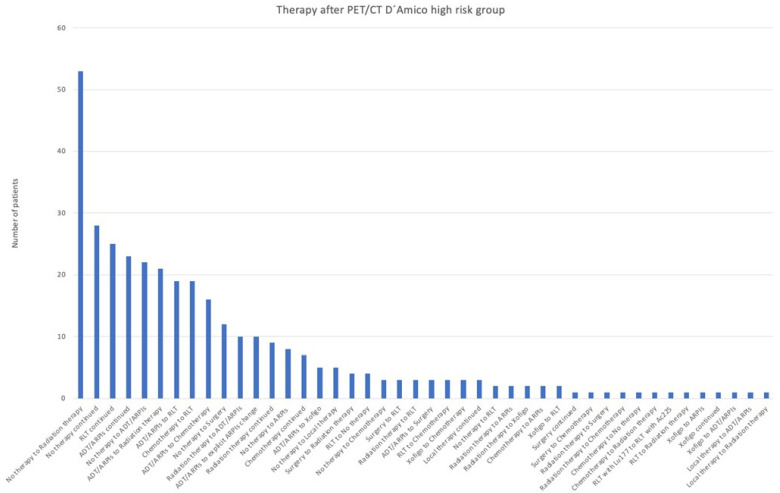
Treatment adjustments by ^68^Ga-PSMA-11-PET/CT in the D’Amico high-risk group.

In the high-risk group, most patients (53/344; 15.4%) were modified from “no therapy” to radiation therapy, 28 (8.1%) continued with no therapy, 25 (7.3%) with RLT, and 23 (6.7%) with ADT/ARPI (Figure 7). There was a treatment change in 22/344 (6.4%) from “no therapy” to ADT/ARPI, in 21/344 (6.1%) from ADT/ARPI to radiation therapy, in 19/344 from chemotherapy and ADT/ARPI to RLT, in 16/344 (4.7%) from ADT/ARPI to chemotherapy, in 12/344 (3.5%) from “no therapy” to surgery, and in 10/344 (2.9%) each from radiation therapy to ADT/ARPI or to an (other) ARPI. Radiation therapy was continued in 9/344 (2.6%), “no therapy” changed to ARPI in 8/344 (2.3%), chemotherapy was continued in 7/344 (2.0%), 5 (1.5%) patients each changed from ADT/ARPI to Xofigo and from “no therapy” to local therapy, and 4 (1.2%) each switched from surgery to radiation therapy and from RLT to “no therapy”. Respectively, three cases (0.9%) were modified from “no therapy” to chemotherapy, from surgery and radiation therapy to RLT, from ADT/ARPI to surgery, and from RLT and Xofigo to chemotherapy, as well as stayed with local therapy. Two (0.6%) patients changed from “no therapy” to RLT, from radiation therapy to ARPI therapy or Xofigo, from chemotherapy to ARPI therapy, and Xofigo to RLT. In 1/344 (0.3%) cases, surgery and Xofigo were continued, surgery changed to chemotherapy, radiation therapy to surgery or chemotherapy, chemotherapy to “no therapy” or radiation therapy, RLT from ^177^Lutetium (^177^Lu) to RLT with ^225^Actinium (^225^Ac) or radiation therapy, Xofigo to ARPI, ADT/ARPI, or was continued, and local therapy to ADT/ARPI or radiation therapy.

### 3.3. Impact of the Gleason Score

**Figure 8:**
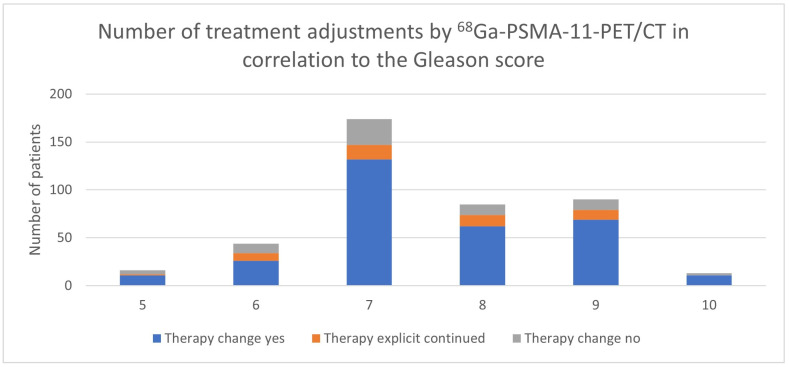
Number of treatment adjustments by ^68^Ga-PSMA-PET/CT in correlation to the Gleason score.

**Figure 9:**
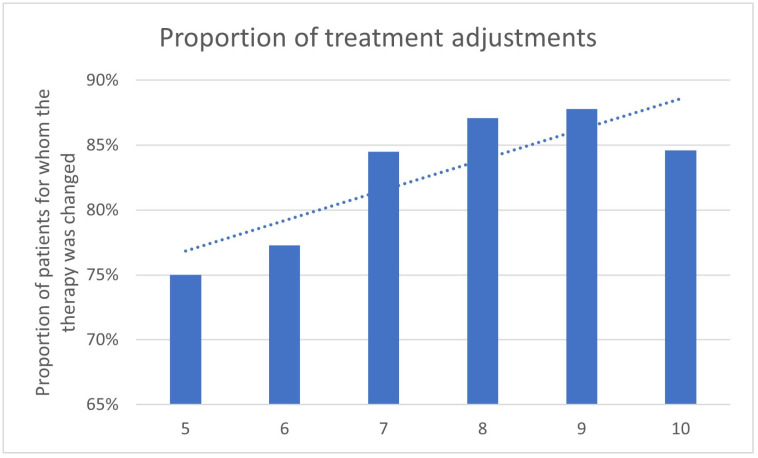
Proportion of treatment adjustments changed by ^68^Ga-PSMA-11-PET/CT in correlation to the Gleason score.

Analyzing the results for the different Gleason scores, it is apparent that with an increasing Gleason score, the proportion of patients who receive a change in therapy as a result of ^68^Ga-PSMA-11-PET/CT appears to increase (Table 4, Figure 8 and Figure 9).

With a Gleason score of 6, the percentage of a treatment change was 77.3%, for a Gleason score of 7, the change was recorded in 84.5%, in patients with a Gleason score of 8, in 87.1%, in patients with a Gleason score of 9, in 87.8%, and in patients with a Gleason score of 10, in 84.6%. The trend line indicates an increased proportion of changed treatment concepts for higher Gleason scores (Figure 9).

The analysis of the Gleason score groups revealed a clear trend, wherein higher scores were significantly associated with an increased likelihood of treatment change. Specifically, patients with Gleason scores of 7 through 10 exhibited statistically significant differences in treatment modification rates (all *p* < 0.01), while no significant association was observed for lower scores (5 and 6; *p*-values > 0.01).

**Figure 10:**
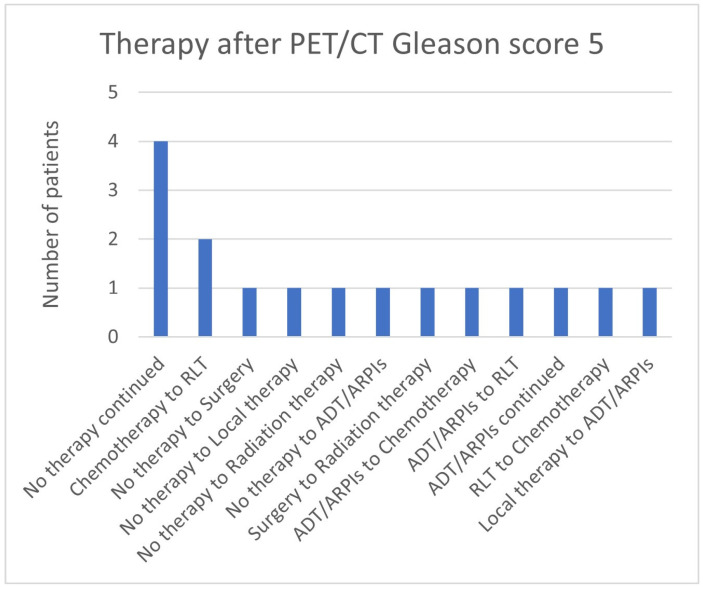
Treatment adjustments by ^68^Ga-PSMA-11-PET/CT in Gleason score 5 group.

In the group with a Gleason score of 5 in 4/14 (28.6%) patients, “no therapy” was continued, 2/14 (14.3%) switched from chemotherapy to RLT, and 1 patient each (7.1%) from “no therapy” to surgery, local therapy, radiation therapy, and ADT/ARPI, as well as from surgery to radiation therapy, RLT to chemotherapy, local therapy to ADT/ARPI, and from ADT/ARPI to chemotherapy, RLT, or continued ADT/ARPI (Figure 10).

**Figure 11:**
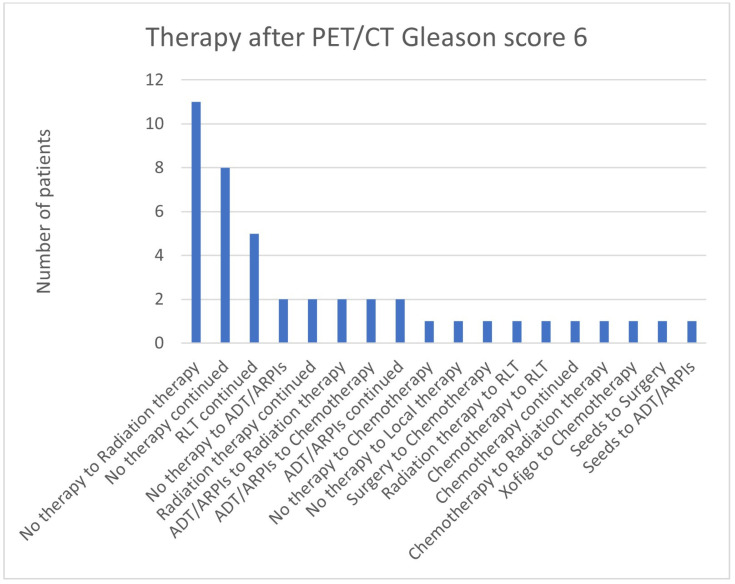
Treatment adjustments by ^68^Ga-PSMA-11-PET/CT in Gleason score 6 group.

In total, 11/44 (25.0%) cases in the group with Gleason scores of 6 changed from “no therapy” to radiation therapy, in 8/44 (18.2%), “no therapy”, and in five (11.4%), RLT was continued (Figure 11). Two patients (4.5%) each went from “no therapy” to ADT/ARPI, ADT/ARPI to radiation therapy and chemotherapy, as well as continued ADT/ARPI and radiation. Respectively, in 1/44 (2.3%) cases, there was a switch from “no therapy” to chemotherapy or local therapy, from surgery to chemotherapy, from radiation therapy and chemotherapy to RLT or radiation therapy or a continuation of the treatment, besides a change from Xofigo to chemotherapy and seeds to surgery or ADT/ARPIs.

**Figure 12:**
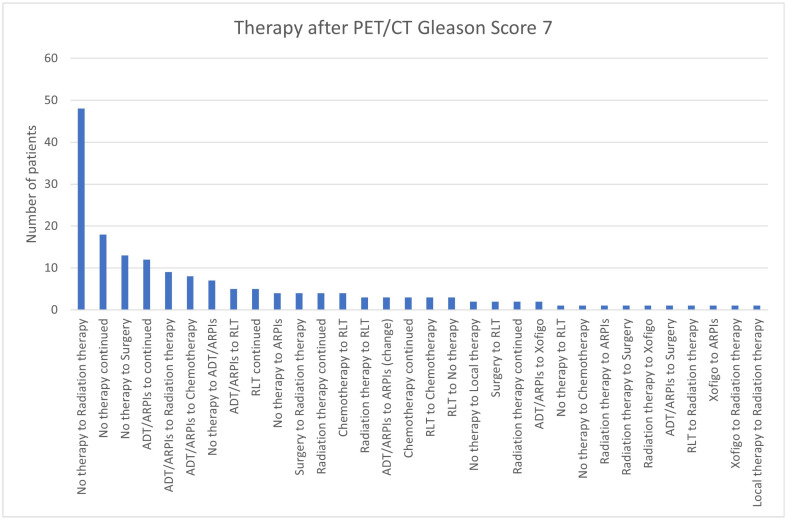
Treatment adjustments by ^68^Ga-PSMA-11-PET/CT in Gleason score 7 group.

For Gleason scores of 7, most patients (48/174; 27.6%) switched from “no therapy” to radiation therapy, “no therapy” was continued in 18/174 (10.3%) cases, 13 (7.5%) changed from “no therapy” to surgery, and 12 (6.9%) continued ADT/ARPI. In total, 9/174 (5.2%) were modified from ADT/ARPI to radiation therapy and 8 (4.6%) to chemotherapy (Figure 12). In total, 5/174 (2.9%) patients switched from “no therapy” to ADT/ARPI, 5 from ADT/ARPI to RLT, and 5 also continued RLT. Four (2.3%) cases each changed from “no therapy” to ARPIs, surgery to radiation therapy, and chemotherapy to RLT or continued radiation therapy, as well as three (1.7%) cases each switched from radiation therapy to RLT, from RLT to chemotherapy or stopped therapy, had an ARPI change, or continued chemotherapy. Respectively, two (1.1%) patients changed from “no therapy” to local therapy, from surgery to RLT, from ADT/ARPI to Xofigo, or continued radiation therapy, and, respectively, one patient went from “no therapy” to RLT and chemotherapy, from radiation therapy to ARPI, to surgery and Xofigo, switched from ADT/ARPI to surgery, RLT to radiation therapy, Xofigo to ARPI, and radiation therapy or local therapy to radiation therapy.

**Figure 13:**
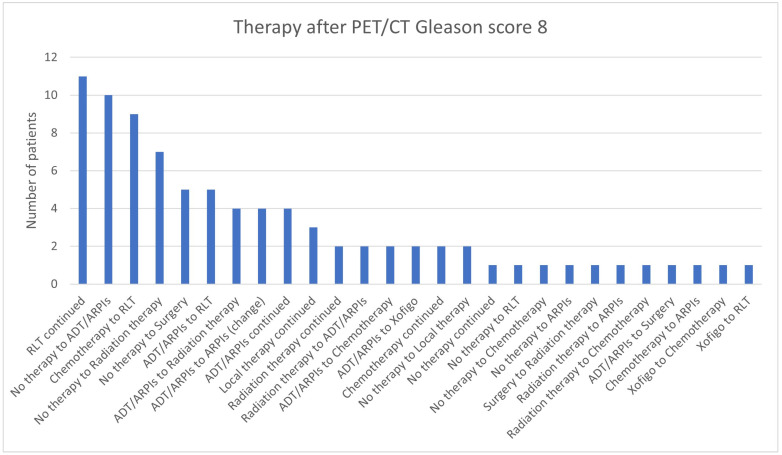
Treatment adjustments by ^68^Ga-PSMA-11-PET/CT in Gleason score 8 group.

In the group with Gleason scores of 8, in 11/85 (35.7%) patients, RLT was continued, 10/85 (11.8%) switched from “no therapy” to ADT/ARPI, 9/85 (10.6%) from chemotherapy to RLT, 9 (10.6%) from chemotherapy to RLT, 7 (8.2%) from “no therapy” to radiation therapy, and 5 (5.9%) patients each from “no therapy” to surgery and ADT/ARPI to RLT (Figure 13). In 4/85 (4.7%) cases, ADT/ARPI was continued or switched to another ARPI or radiation therapy. In three patients, local therapy was continued. Two cases each continued radiation therapy or chemotherapy, changed from radiation therapy to ADT/ARPI, or from ADT/ARPI to chemotherapy and Xofigo. Respectively, one (1.2%) patient went from “no therapy” to RLT, chemotherapy, or ARPI, from surgery to radiation therapy, from radiation therapy to ARPI or chemotherapy, from ADT/ARPI to surgery, from chemotherapy to ARPI, or from Xofigo to chemotherapy or RLT.

**Figure 14:**
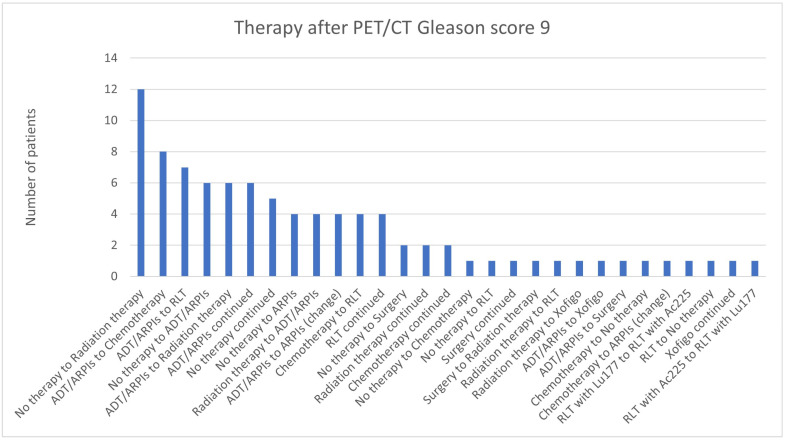
Treatment adjustments by ^68^Ga-PSMA-11-PET/CT in Gleason score 9 group.

For Gleason scores of 9, most patients (12/90; 13.3%) switched from “no therapy” to radiation therapy, 8/90 (8.9%) from ADT/ARPI to chemotherapy, or seven (7.8%) to RLT (Figure 10). In six (6.7%) cases, there was a change from “no therapy” to ADT/ARPI, ADT/ARPI to radiation therapy, or an ADT/ARPI continuation, and in five (5.6%), “no therapy” was continued (Figure 14). In total, 4/90 (4.4%) patients each were modified from “no therapy” to ARPIs, radiation therapy to ADT/ARPI, ADT/ARPI to ARPI change, chemotherapy to RLT, or RLT was continued. Two cases were modified from “no therapy” to surgery or continued radiation therapy or chemotherapy. In one (1.1%) patient, there was surgery or Xofigo continued, or a modification from “no therapy” to chemotherapy, “no therapy” to RLT, surgery to radiation therapy, radiation therapy to RLT or Xofigo, ADT/ARPI to Xofigo or surgery, chemotherapy to “no therapy” or ARPI, RLT with ^177^Lutetium to RLT with ^225^Actinium, RLT with ^225^Actinium to RLT with ^177^Lutetium, or therapy was stopped.

**Figure 15:**
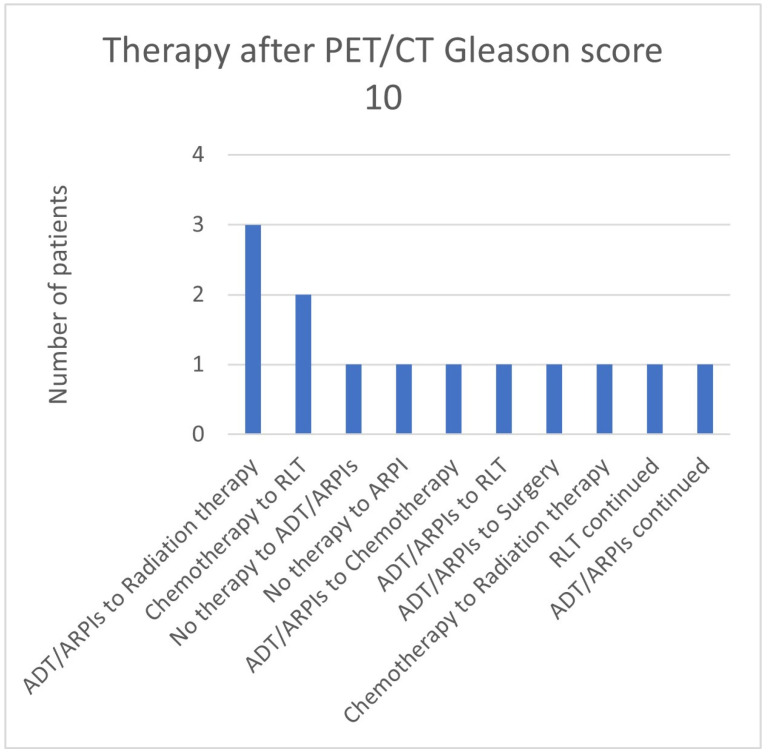
Treatment adjustments by ^68^Ga-PSMA-11-PET/CT in Gleason score 10 group.

In the group with Gleason scores of 10, in 3/13 (23.1%) patients, ADT/ARPI was changed to radiation therapy, in two (15.4%), chemotherapy was changed to RLT, and in one (7.7%) patient each, “no therapy” was switched to ADT/ARPI or ARPI, ADT/ARPI to chemotherapy or RLT or surgery, chemotherapy to radiation therapy, and RLT or ADT/ARPI were continued (Figure 15).

### 3.4. Impact of PSA Value

**Figure 16:**
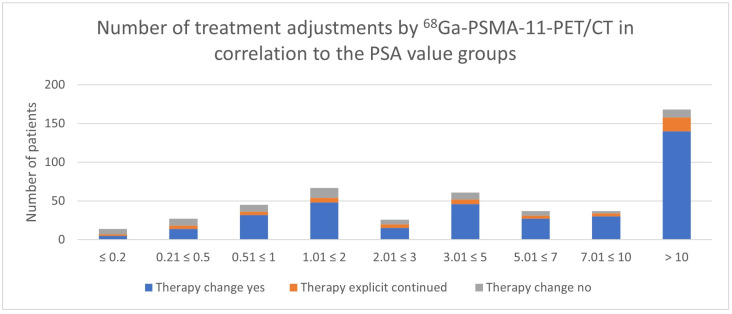
Number of treatment adjustments by ^68^Ga-PSMA-11-PET/CT in correlation to the PSA value.

**Figure 17:**
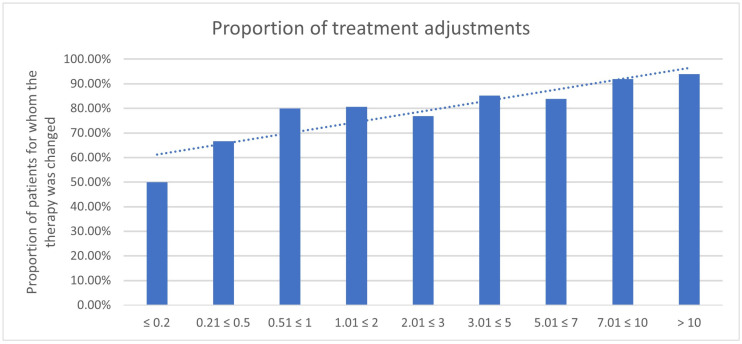
Proportion of treatment adjustments changed by ^68^Ga-PSMA-11-PET/CT in correlation to the PSA value.

Regarding the results of the PSA value impact, there is a tendency for the ^68^Ga-PSMA-11-PET/CT to lead to a more frequent adjustment in treatment management with increasing PSA levels (Table 5, Figure 16 and Figure 17). The proportion of a treatment change was 50.0% for a PSA value of ≤0.2 ng/mL; 66,7% for a PSA value of 0.21 ≤ 0.5 ng/mL; 80.0% for a PSA value of 0.51 ≤ 1 ng/mL; 80.6% for a PSA value of 1.01 ≤ 2 ng/mL; 67.9% for a PSA value of 2.01 ≤ 3 ng/mL; 85.2% for a PSA value of 3.01 ≤ 5 ng/mL; 83.8% for a PSA value of 5.01 ≤ 7 ng/mL; 91.9% for a PSA value of 7.01 ≤ 10 ng/mL; and 94.0% for a PSA value of >10 ng/mL.

The analysis of the PSA-level groups showed that elevated PSA values were correlated with a higher probability of treatment change. This association was statistically significant for PSA levels above 0.5 ng/mL, particularly within the ranges of 1.01 ≤ 2 ng/mL (*p* = 0.0002) and 3.01 ≤ 5 ng/mL (*p* < 0.0001) and values exceeding 10 ng/mL (*p* < 0.0001).

**Figure 18:**
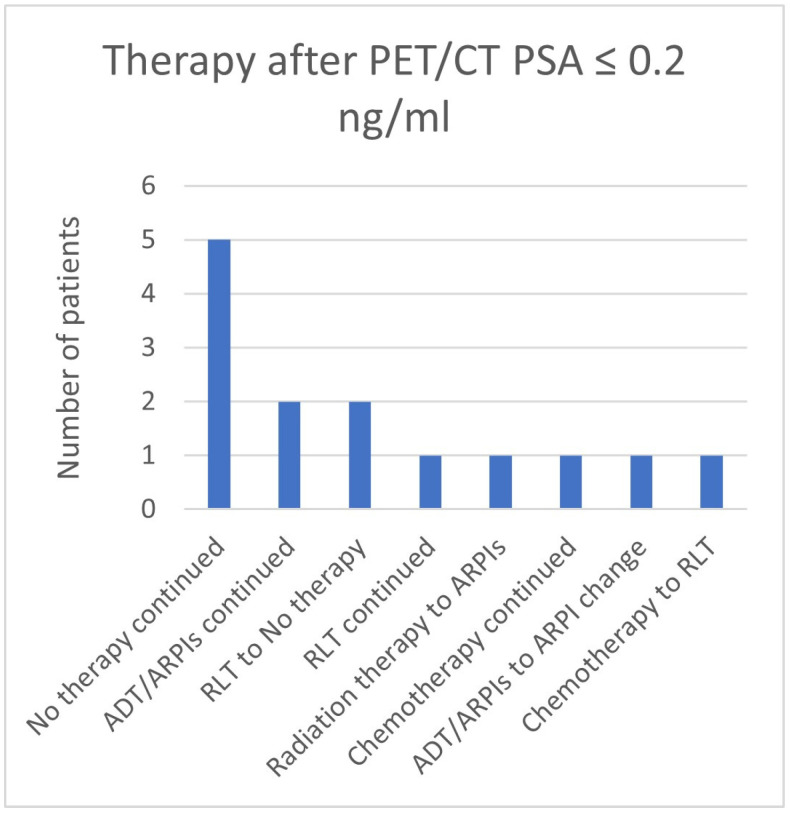
Treatment adjustments by ^68^Ga-PSMA-11-PET/CT in the PSA group, PSA ≤ 0.2 ng/mL.

In the group with a PSA value ≤ 0.2 ng/mL, in 5/14 (35.7%) patients, “no therapy” was continued, in 2/14 (13.3%), therapy with ADT/ARPIs was continued, in 2 cases, therapy changed from RLT to no therapy, and in, respectively, 1/14 (7.1%) cases, RLT was continued, radiation therapy changed to ARPI, chemotherapy was continued, ARPI was changed, and chemotherapy switched to RLT (Figure 18).

**Figure 19:**
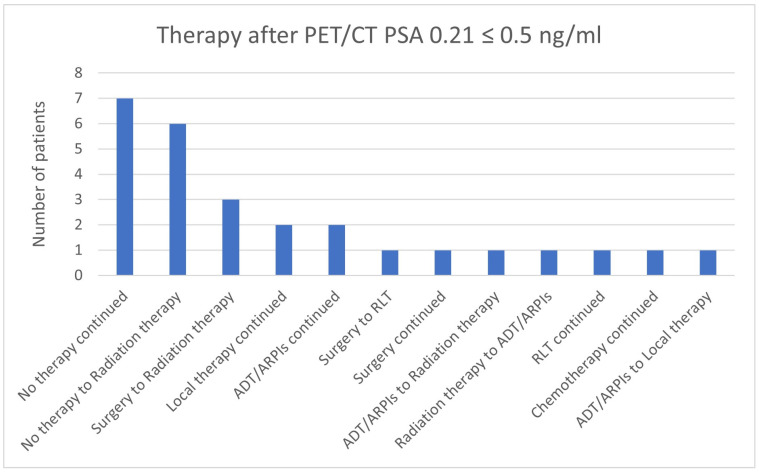
Treatment adjustments by ^68^Ga-PSMA-11-PET/CT in the PSA group, PSA of 0.21 ≤ 0.5 ng/mL.

In the group with a PSA value of 0.21 ≤ 0.5 ng/mL, in 7/27 (25.9%) patients, “no therapy” was continued, “no therapy” was changed to radiation therapy in 6/27 (22.2%) patients, surgery was switched to radiation therapy in 3/27 (11.1%) cases, local therapy and ADT/ARPIs were continued in 2/27 (7.4%) patients each and in, respectively, 1/27 (3.7%) cases, surgery was changed to RLT, surgery was continued, ADT/ARPIs were modified to radiation therapy, radiation therapy to ADT/ARPI, ADT/ARPI to local therapy and RLT, and chemotherapy continued (Figure 19).

**Figure 20:**
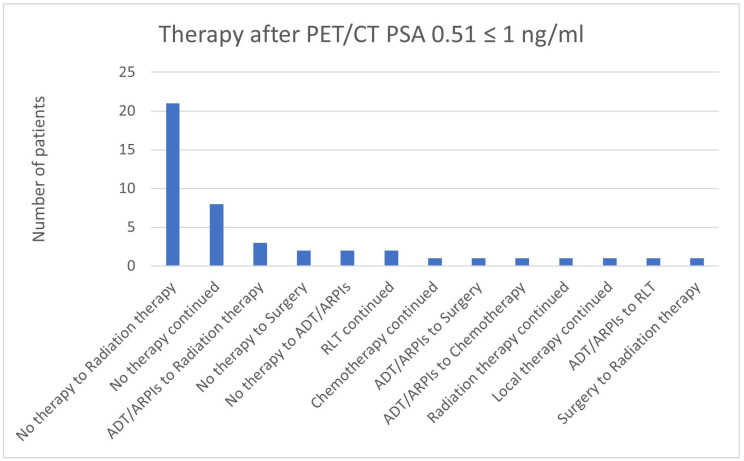
Treatment adjustments by ^68^Ga-PSMA-11-PET/CT in the PSA group, PSA of 0.51 ≤ 1 ng/mL.

In total, 21/45 (46.7%) cases in the group with a PSA value of 0.51 ≤ 1 ng/mL changed from “no therapy” to radiation therapy, in 8/45 (17.8%) patients, “no therapy” was continued, and 3/45 (6.7%) switched from ADT/ARPIs to radiation therapy (Figure 20). Two patients each (2/45; 4.4%) went from “no therapy” to surgery and ADT/ARPIs, as well as continued RLT. In, respectively, 1/45 (2.2%) cases, chemotherapy, radiation therapy, and local therapy were continued, as well as the change from ADT/ARPIs to surgery, to chemotherapy, and to RLT, and surgery to radiation therapy.

**Figure 21:**
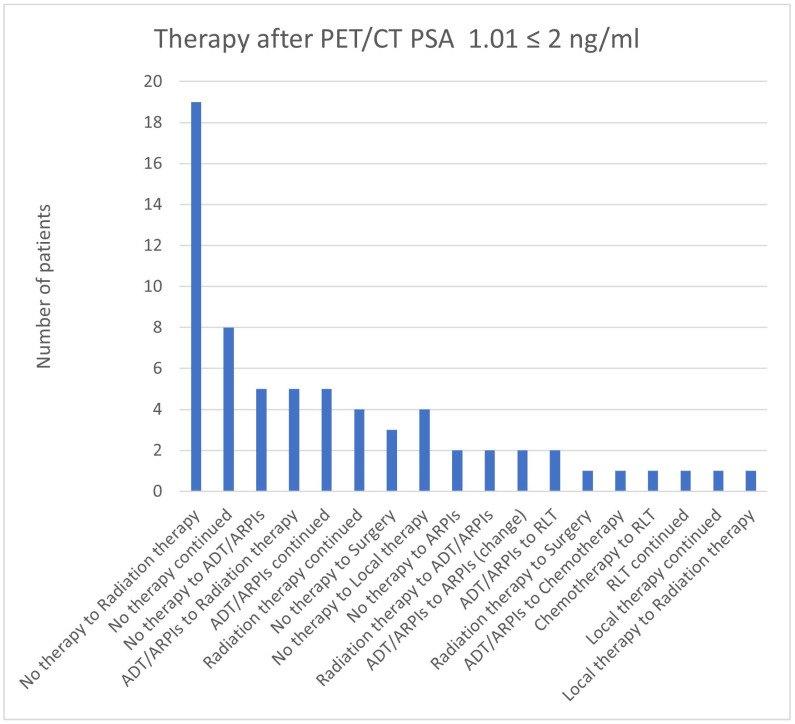
Treatment adjustments by ^68^Ga-PSMA-11-PET/CT in the PSA group, PSA of 1.01 ≤ 2 ng/mL.

For PSA values of 1.01 ≤ 2 ng/mL, 19/67 (28.4%) patients changed from “no therapy” to radiation therapy, and in 8/67 (11.9%) patients, “no therapy” was continued (Figure 21). In total, 5/67 (7.5%) cases were modified from “no therapy” to ADT/ARPIs and ADT/ARPIs to radiation therapy, as well as continued ADT/ARPIs. Four (6.0%) patients switched from “no therapy” to local therapy or continued radiation, and three (4.5%) from “no therapy” to surgery. In total, 2/67 (3.0%) had a change from “no therapy” to ARPI, radiation therapy to ADT/ARPIs, an ARPI change, or ADT/ARPIs to RLT. In, respectively, 1/67 (1.5%) cases, radiation therapy changed to surgery, ADT/ARPIs to chemotherapy, chemotherapy to RLT, local therapy to radiation therapy or RLT, and local therapy were continued.

**Figure 22:**
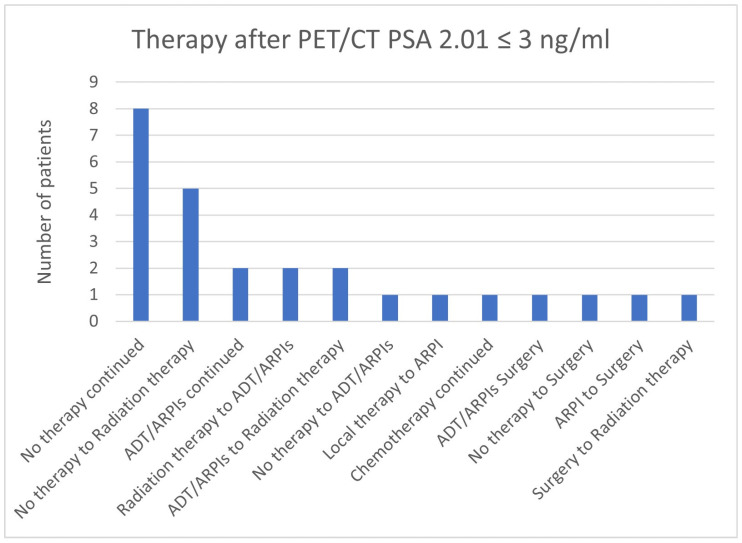
Treatment adjustments by ^68^Ga-PSMA-11-PET/CT in the PSA group, PSA of 2.01 ≤ 3 ng/mL.

In total, 8/26 (30.8%) cases in the group with a PSA value of 2.01 ≤ 3 ng/mL continued “no therapy”, 5/26 (19.2%) switched from “no therapy” to radiation therapy, 2 (7.7%) each from radiation therapy to ADT/ARPIs and ADT/ARPIs to radiation therapy (Figure 22). In, respectively, 1/26 (3.8%) cases, “no therapy” or local therapy was modified to ADT/ARPI; ADT/ARPI, ARPI, or “no therapy” to surgery, surgery to radiation therapy, or chemotherapy continued.

**Figure 23:**
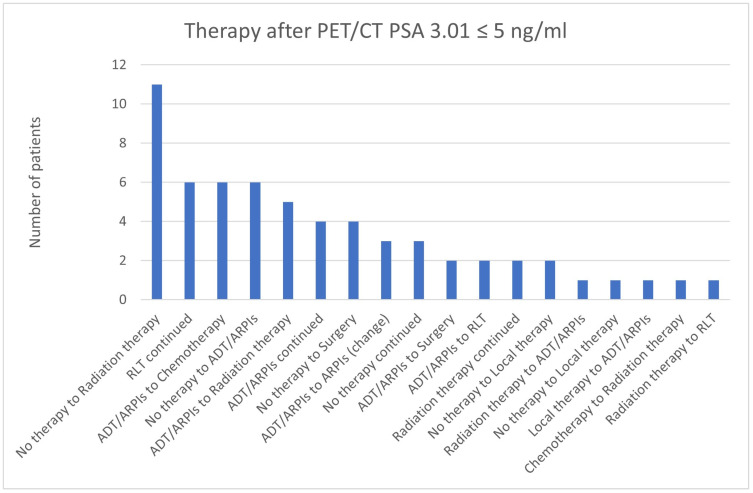
Treatment adjustments by ^68^Ga-PSMA-11-PET/CT in the PSA group, PSA of 3.01 ≤ 5 ng/mL.

For PSA values of 3.01 ≤ 5 ng/mL, 11/61 (18.0%) patients changed from “no therapy” to radiation therapy. In 6/61 (9.8%) patients, “no therapy” was switched to ADT/ARPI and ADT/ARPI to chemotherapy, as well as RLT continued (Figure 23). In 5/61 (8.2%) cases, ADT/ARPI was modified to radiation therapy, in 4/61 (6.6%), “no therapy” to surgery, and ADT/ARPI continued. The ADT/ARPI to ARPI change was found in 3/61 (4.9%), and the same percentage continued with “no therapy”. In 2/61 (3.3%) patients, each ADT/ARPI was changed to surgery and RLT, “no therapy” to local therapy, and radiation therapy continued. In, respectively, 1/61 (1.6%) cases, radiation therapy switched to ADT/ARPI, “no therapy” to local therapy, local therapy to ADT/ARPI, chemotherapy to radiation therapy, and radiation therapy to RLT.

**Figure 24:**
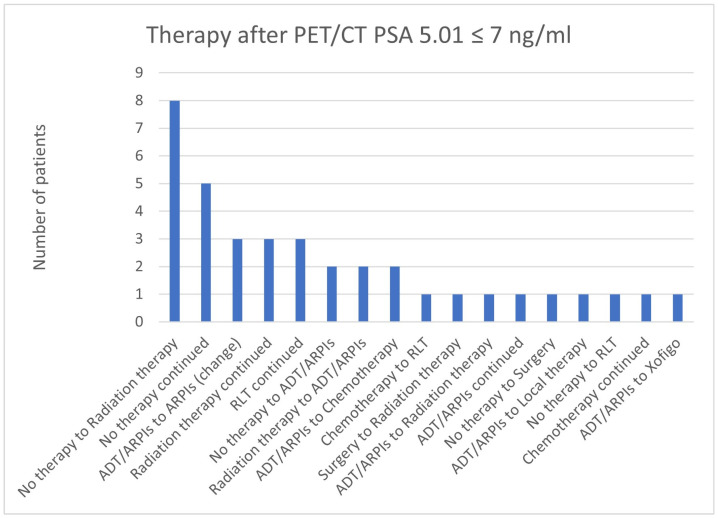
Treatment adjustments by ^68^Ga-PSMA-11-PET/CT in the PSA group, PSA of 5.01 ≤ 7 ng/mL.

In total, 8/37 (21.6%) cases in the group with a PSA value of 5.01 ≤ 7 ng/mL switched from “no therapy” to radiation therapy, 5/37 (13.5%) continued with “no therapy”, 3/37 (8.1%) each continued radiation therapy, RLT, or started/changed the ARPI (Figure 24). In total, 2/37 (5.4%) started with ADT/ARPI and changed from radiation therapy to ADT/ARPI or from ADT/ARPI to chemotherapy. In 1/37 (2.7%) cases each, there was a change from chemotherapy to RLT, surgery, or ADT/ARPI to radiation therapy, “no therapy” to surgery or RLT, ADT/ARPI to local therapy or Xofigo, and continued ADT/ARPI or chemotherapy.

**Figure 25:**
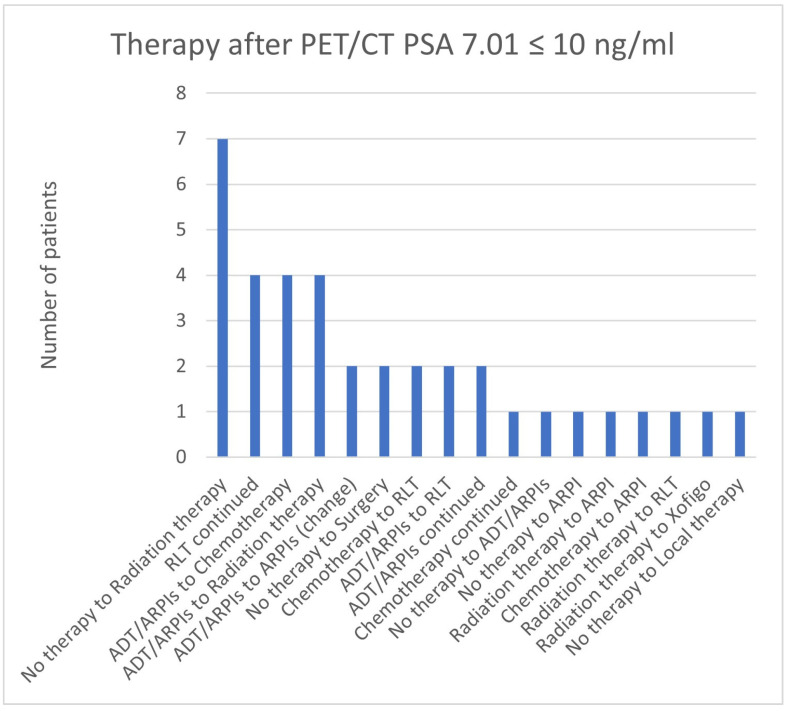
Treatment adjustments by ^68^Ga-PSMA-11-PET/CT in the PSA group, PSA of 7.01 ≤ 10 ng/mL.

For PSA values of 7.01 ≤ 10 ng/mL, 7/37 (18.9%) patients changed from “no therapy” to radiation therapy, in 4/37 (10.8%), RLT was continued, and ADT/ARPI changed to chemotherapy or radiation therapy (Figure 25). In total, 2/37 (5.4%) cases, respectively, had an ARPI change, a modification from “no therapy” to surgery, from chemotherapy or ADT/ARPI to RLT, and continued ADT/ARPI therapy. In total, 1/37 (2.7%) cases showed a change from “no therapy” to ADT/ARPI and ARPI, a chemotherapy continuation, a modification from radiation therapy or chemotherapy to ARPI, radiation therapy to RLT or Xofigo, and “no therapy” to local therapy.

**Figure 26:**
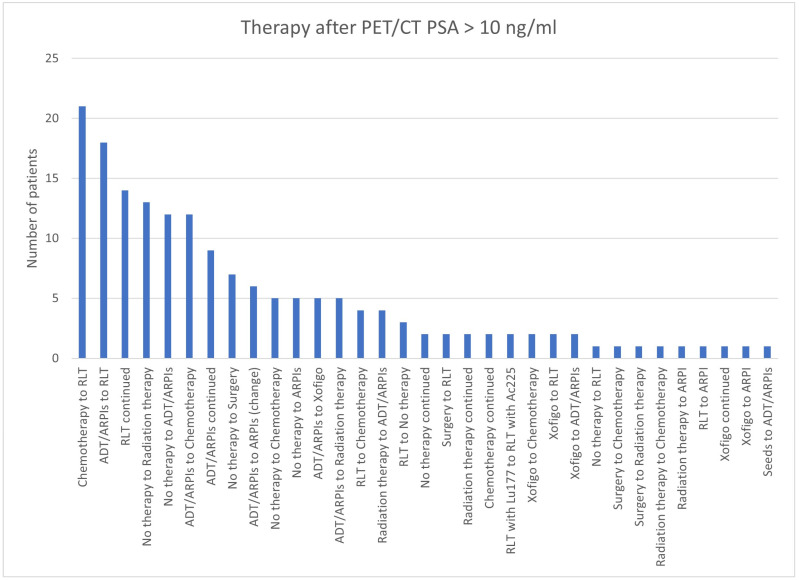
Treatment adjustments by ^68^Ga-PSMA-11-PET/CT in PSA group, PSA > 10 ng/mL.

In total, 21/168 (12.5%) cases in the group with a PSA value > 10 ng/mL switched from chemotherapy to RLT, 18/168 (10.7%) changed from ADT/ARPI to RLT, 14/168 (8.3%) continued RLT; from “no therapy”, 13 (7.7%) switched to radiation therapy and 12 (7.1%) to chemotherapy (Figure 26). In 9/168 (5.4%), ADT/ARPI was continued, in 7 (4.2%), a change from “no therapy” to surgery was found, and in 6 (3.6%), a change in the ARPI. In total, 5/168 (3.0%) patients each switched from “no therapy” to chemotherapy and ARPI treatment or from ADT/ARPI to Xofigo and radiation therapy. Four (2.4%) showed a modification from RLT to chemotherapy and from radiation therapy to ADT/ARPI. In three (1.8%) patients, RLT was stopped to “no therapy”. In 2/168 (1.2%) patients, a modification could be registered from surgery to RLT, RLT with ^177^Lu to RLT with ^225^Ac, Xofigo to chemotherapy, RLT and ADT/ARPI, and “no therapy”, radiation therapy, and chemotherapy were continued. Respectively, in one (0.6%) patient, there was a change from “no therapy” to RLT, surgery to chemotherapy and radiation therapy, radiation therapy to chemotherapy and ARPI, RLT to ARPI, seeds to ADT/ARPI, Xofigo to ARPI, or a Xofigo continuation.

### 3.5. Continuation of Therapy Based on the ^68^Ga-PSMA-PET/CT Findings

**Figure 27:**
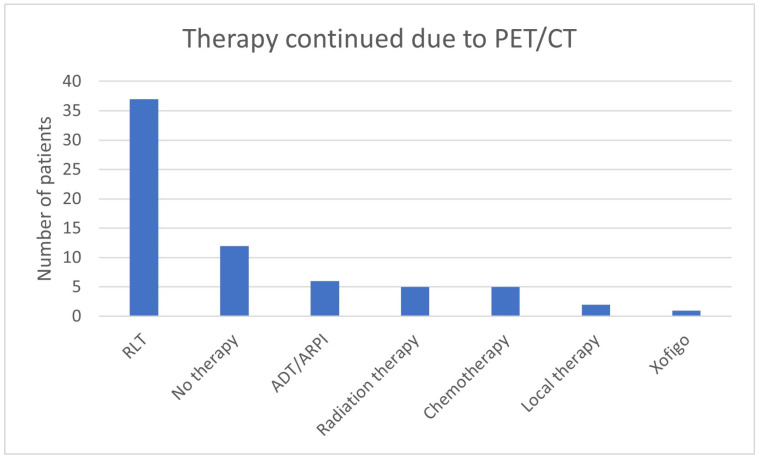
Therapy explicitly continued based on the ^68^Ga-PSMA-11-PET/CT findings.

**Figure 28:**
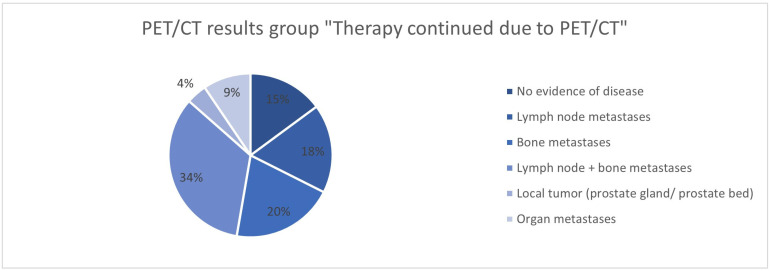
^68^Ga-PSMA-11-PET/CT findings in the group “Therapy continued due to PET/CT”.

Therapy was continued due to the ^68^Ga-PSMA-11-PET/CT findings in 68/562 (12.1%) of all cases and in 68/147 (46.3%) cases in which therapy was continued explicitly for this reason (Table 6, Figure 27). In 37/68 (54.4%), the therapy that was continued was RLT, in 12/68 (17.6%), “no therapy” was continued, in 6/68 (8.8%), ADT/ARPI was continued, and in 5/68 (7.4%) each, radiation therapy and chemotherapy was continued (Figure 28). In two patients (2.9%), local therapy, and in one patient (1.5%), Xofigo was maintained. There were no clear differences between the groups in which the various therapies were continued. As was to be expected clinically, the further escalated treatment options (RLT and chemotherapy) showed more advanced findings in metastases. A Gleason score of 9, D’Amico high risk, and a higher PSA value were also found in patients who only received continuation of radiotherapy, ADT/ARPI, or “no therapy”.

The groups that received either a continuation of therapy without reference to the examination or with explicit reference to the investigation and the group in which the therapy was modified as a result are shown in Table 7. There are no distinct differences between the three groups with regard to the PSA value, Gleason score, or D’Amico risk classification.

**Figure 29:**
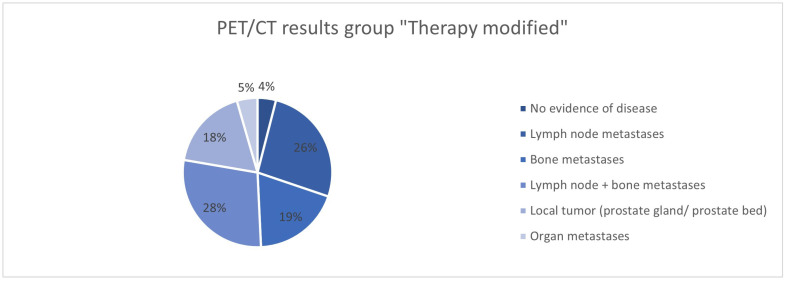
^68^Ga-PSMA-11-PET/CT findings in the group “Therapy modified”.

**Figure 30:**
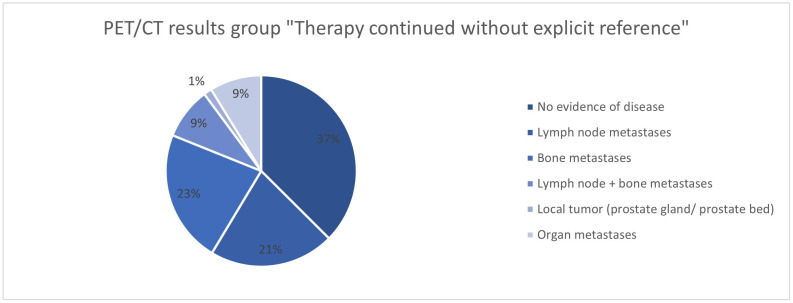
^68^Ga-PSMA-11-PET/CT findings in the group “Therapy continued without explicit reference”.

The analysis of the ^68^Ga-PSMA-11-PET/CT findings in the groups “Therapy continued due to PET/CT”, “Therapy modified”, and “Therapy continued without explicit reference” shows differences in the groups. While no evidence of the disease was found in 38% of cases in the “Therapy continued without explicit reference” group, this was significantly less common in the “Therapy continued due to PET/CT” group (16.2%) and the “Therapy modified” group (4.3%) (Figure 28, Figure 29 and Figure 30). In the group with the adapted therapy, there was a significantly higher proportion of local tumors (19.2%) compared to the other groups (4.4% and 1.4%); organ metastases were found least frequently here (4.8% vs. 10.3% and 8.9%). The distribution of patients with lymph node and/or bone metastases is comparable in all groups.

### 3.6. Therapies Based on the ^68^Ga-PSMA-PET/CT Findings in Low- and Intermediate-Risk Patients

We additionally analyzed the changes in therapy with regard to the PET/CT findings in low- and intermediate-risk patients (Table 8 and Table 9). 

In the low-risk group, in one patient with bone and organ metastases, ADT/ARPI was continued, and in another one with bone metastases, radiation therapy was continued (Table 8). Of the six patients with local tumor detection, four changed from no therapy to radiation therapy, one continued with no therapy, and one changed from seeds to surgery. One patient each with lymph node and bone metastases was modified from chemotherapy to RLT, and the other one from seeds to systemic treatment with ADT/ARPI. Of the five patients with lymph node metastases, three continued with no therapy, one changed from no therapy to ADT/ARPI, and one from chemotherapy to radiation therapy. The one patient with lymph node, bone, and organ metastases was modified from ADT/ARPI to chemotherapy.

In the intermediate-risk group, three of the five patients with bone metastases continued RLT, and one patient each switched from no therapy to chemotherapy or radiation therapy (Table 9). Both patients with local tumor detection were modified from no therapy to radiation therapy. Of those who had lymph node and bone metastases, both patients were switched to a systemic approach: one patient received ADT/ARPI, and the other one was modified from radiation therapy to RLT. In the three patients with only lymph node metastases, one each changed from surgery to radiation therapy, and radiation therapy or ADT/ARPI were continued. The one patient with lymph node metastases and local tumor detection received an initiation of radiation therapy. Of the four patients with no tumor lesion imageable, three continued with no therapy, and one received continued radiation therapy.

### 3.7. Therapies Based on the ^68^Ga-PSMA-PET/CT Findings in Patients with a PSA ≤ 2 ng/mL

Furthermore, we analyzed therapies after PET/CT imaging with regard to the findings in patients with low PSA values (Table 10, Table 11, Table 12 and Table 13).

In the group with a PSA ≤ 0.2 ng/mL, in one patient, radiation therapy was changed to ARPI, and in one patient each, ADT/ARPI or no therapy was continued. In the one patient with local tumor detection, ADT/ARPI was continued. Of the five patients with lymph node metastases, one patient each continued with no therapy, RLT, or chemotherapy, or switched from chemotherapy to RLT or from RLT to no therapy. In the three patients without an imageable tumor lesion, one changed from RLT to no therapy, three continued with no therapy, and one continued ADT/ARPI.

In the group with a PSA of 0.21 ≤ 0.5 ng/mL, one patient with bone metastases continued local therapy, and one changed from no therapy to radiation therapy. Of the four patients with local tumor detection, one patient each continued surgery, changed from ADT/ARPI to local therapy, or from no therapy and surgery to radiation therapy. In the group with lymph node and bone metastases, one patient continued RLT, one continued chemotherapy, and the third one continued ADT/ARPI. Of those with only lymph node metastases, three went from no therapy to radiation therapy and one from surgery to RLT. Twelve patients had no tumor lesion imageable: seven of them continued with no therapy, one with ADT/ARPI, and two changed from surgery, one from no therapy and one from ADT/ARPI to radiation therapy. Of the two patients with organ metastases, one continued with local therapy; the second was modified from radiation therapy to ADT/ARPI.

In the group with a PSA of 0.51 ≤ 1 ng/mL and bone metastases, two patients continued RLT, one continued chemotherapy and one local therapy, as well as one patient each changed from no therapy to ADT/ARPI, one from ADT/ARPI to chemotherapy, and one from surgery or no therapy to radiation therapy. Of those with local tumor detection, all six patients changed to radiation therapy, four from no therapy and two from ADT/ARPI. One patient each with lymph node and bone metastases continued with radiation therapy or changed from ADT/ARPI to radiation therapy or from no therapy to ADT/ARPI. Of the 18 patients with lymph node metastases, 13 changed from no therapy to radiation therapy, 2 each from no therapy to surgery or continued with no therapy, and 1 was switched from ADT/ARPI to RLT. Of the nine patients with no imageable tumor lesion, six continued with no therapy, two received radiation therapy instead of proceeding with no therapy, and one changed from ADT/ARPI to surgery. The one patient with organ metastases was modified from no therapy to radiation therapy.

Regarding the group with a PSA of 1.01 ≤ 2 ng/mL, of the seventeen patients, five were modified from no therapy to ARPI, three from no therapy to radiation therapy, three from radiation therapy to ADT/ARPI, two continued RLT or no therapy, and one patient each continued local therapy or changed from local therapy to radiation therapy. Of those with local tumor detection, two patients were modified from no therapy to local therapy or to radiation therapy, and one patient each from no therapy to surgery or radiation therapy or from ADT/ARPI to radiation therapy. The one patient with local tumor detection and organ metastases was switched from no therapy to radiation therapy. One patient each with lymph node and bone metastases continued local therapy, changed from no therapy to radiation therapy or ADT/ARPI, or from ADT/ARPI to radiation therapy. The only patient in this group with lymph node and bone metastases, as well as local tumor detection, was switched from radiation therapy to ADT/ARPI. Of the twenty-six patients with only lymph node metastases, seven were modified from ADT/ARPI to radiation therapy, three each continued ADT/ARPI or changed from ADT/ARPI to chemotherapy or from no therapy to local therapy. Two each switched from ADT/ARPI or chemotherapy to RLT, and one patient each from no therapy to ADT/ARPI or surgery or radiation therapy, or from ADT/ARPI to another ARPI, or continued radiation therapy. Two of the four patients with lymph node metastases and local tumor detection went from no therapy to radiation therapy, one from no therapy to local therapy, and one continued ADT/ARPI. The one patient with lymph node, bone, and organ metastases was modified from ADT/ARPI to RLT. Of the six patients with no imageable tumor lesion, two changed from no therapy to radiation therapy, one from no therapy to ADT/ARPI, and one each continued no therapy or radiation therapy or ADT/ARPI therapy.

## 4. Discussion

^68^Ga-PSMA-PET/CT is established in the clinical routine of prostate cancer diagnostics and follow-up with studies showing an excellent sensitivity and specificity, especially in patients with biochemical recurrence, but also for therapy monitoring [1].

The findings of this study indicate that treatment modification in patients with prostate cancer is significantly associated with specific clinical parameters. According to the analysis based on the D’Amico risk classification, only patients categorized as high risk demonstrated a statistically significant association with treatment change (*p* < 0.0001), whereas no significant associations were observed in the low- and intermediate-risk groups (Table 3, Figure 3).

An analysis of the Gleason score revealed a clear trend, wherein higher scores were significantly associated with an increased likelihood of treatment change. Specifically, patients with Gleason scores of 7 through 10 exhibited statistically significant differences in treatment modification rates (all *p* < 0.01), while no significant association was observed for lower scores (5 and 6).

Some studies show correlations between the D’Amico risk classification groups and the Gleason score with PET/CT results. In a recent study by Ulas Babacan et al., there was a strong, statistically significant correlation (*p* < 0.0001) between the D’Amico risk classification, Gleason score, and the presence or absence of metastasis, as well as between the median SUVmax value of the prostate gland and the D’Amico risk classification [8].

Similarly, an analysis of PSA levels showed that elevated PSA values were correlated with a higher probability of treatment change. This association was statistically significant for PSA levels above 0.5 ng/mL, particularly within the ranges of 1.01 ≤ 2 ng/mL (*p* = 0.0002), 3.01 ≤ 5 ng/mL (*p* < 0.0001), 5.01 ≤ 10 ng/mL (*p* = 0.0001), and values exceeding 10 ng/mL (*p* < 0.0001).

Van Leeuwen et al. studied 70 patients and observed that in 28.6%, ^68^Ga-PSMA-PET/CT led to a major change in treatment management [9]. Morigi et al. were able to show, with a comparison between ^18^Fluorine (^18^F)-Fluoromethylcholine and ^68^Ga-PSMA-PET/CT in 38 patients, a change in the treatment plan in 63% of patients after PET/CT diagnostics, and a significantly higher detection rate for ^68^Ga-PSMA-PET/CT imaging than for ^18^F-Fluoromethylcholine [10]. Shakespeare showed a change in treatment plans in 53.7% of the cases investigated [11]. He included 54 patients who received PSMA-PET/CT for staging before radiation therapy as a definitive (14.8%) or post-prostatectomy (33.3%) therapy, and in the presence of PSA failures following definitive (16.7%) or post-prostatectomy (33.3%) radiotherapy. In a current study focusing on therapy planning in radiation oncology, Bock et al. demonstrated an impact of PSMA-PET/CT on salvage radiation therapy planning in almost 63% and for definitive radiation therapy in 9% of patients by a change in the planning target volume and additional boosts [12].

In our study, the high proportion of “No evidence of disease” in the group “Therapy continued without explicit reference” can be clinically concluded, also with regard to the high proportion of “No evidence of disease” in patients who continued “no therapy” due to the PET/CT examination.

The high proportion of “Local tumor” in the group of adapted therapies can be clinically derived from the patients who switched from “no therapy” to radiotherapy or surgery. These correlations are shown in various studies with regard to therapy modification prior to radiotherapy and/or surgery.

In a prospective Australian multicenter study with 431 patients, ^68^Ga-PSMA-PET/CT was performed for restaging or biochemical recurrence in 75% of patients and for the primary staging of intermediate- and high-risk disease in 25% of patients [13]. Overall, the ^68^Ga-PSMA-PET/CT scan led to a change in planned treatment in 51% of patients. The impact was smaller in the group undergoing primary staging (21% change) than in patients with biochemical failure after definitive surgery or radiation, with a 62% change in treatment. Imaging with ^68^Ga-PSMA-PET/CT revealed locoregional lymph nodes in 39%, disease in the prostate bed in 27%, and distant metastases in 16% of patients.

Hope et al. reported a treatment change rate for patients with biochemical recurrence of 53% [14]. Calais et al. found a 61% change rate in 2018, and in a large prospective study in 2020, Fendler et al. were able to show an intended management change in 68% of patients, with an intended change considered as major in 46% of patients. Those major changes occurred most often (55%) in patients with PSA values of 0.5 ng/mL to less than 2.0 ng/mL [15,16]. Our findings, with 73.8% treatment changes, confirm this trend with increasing importance, which the studies have shown over the years. A 2018 meta-analysis involving 1163 patients reported a 54% (95% confidence interval, 47–60%) treatment change after ^68^Ga-PSMA-PET/CT [3]. Compared to the meta-analysis by Han et al., which involved studies with smaller sample sizes, the number of patients ranged from 15 to 150 per study; our analysis reflects higher change rates in a larger cohort, including 562 cases [3]. Sterzing et al. reported a 50.8% change in the TNM stage among 57 patients, with subsequent changes in treatment management [17]. In a similar study, 53% of patients had a TNM-stage change, and 33.3% adjusted their radiotherapy based on ^68^Ga-PSMA-PET/CT results [18]. In contrast, Calais et al. reported only a 16.5–37% influence of ^68^Ga-PSMA-PET/CT on definitive radiotherapy [19]. Schmidt-Hegemann et al. documented a 50% change in treatment plans for patients with PSA recurrence and noted significant differences in radiotherapy procedures compared to CT alone, especially postoperatively [20]. Their study from 2017 indicated a 56% adaptation rate, while another showed a 62% change in radiotherapy concepts in retrospectively examined cases [20,21].

Additionally, we analyzed a change in all available treatment options, such as uro-oncological and radiotherapeutic treatment concepts, such that direct comparisons with purely radiotherapy studies are limited. Notably, other more recent studies, as performed by Schmidt-Hegemann et al., showed higher adaptation rates (53% in 2017 and 62% in 2019) compared to older studies (33.3% in Dewes et al., 2016), reflecting progress in treatment strategies [18,21,22]. It is important to recognize not just the rate of therapy changes but also how ^68^Ga-PSMA-PET/CT enables advanced techniques like image-guided radiotherapy [23].

Interestingly, the risk of recurrence increased in a study by King by 2.6% for every 0.1 ng/mL rise in PSA, suggesting that salvage therapy is most effective at lower PSA levels [24]. This may explain the high treatment change rates in patients with lower PSA values, although our analysis showed that a treatment decision based on PET/CT is made in up to 80.6% of cases with a PSA ≤ 2 ng/mL. This rate is distinctly higher than reported by King et al., though their focus was on salvage radiotherapy, making direct comparisons difficult.

Sonni et al. were able to reveal an effect on the treatment in 104/182 (57%) of the included patients [25]. PSMA-PET/CT influenced clinical management in 72% (13/18) of patients restaged after radiation therapy (not reaching a biochemical recurrence), 67% (8/12) of those evaluated following other definitive local treatments, and 61% (59/96) of patients with advanced metastatic disease.

Due to the high sensitivity (pooled 71–80%) and specificity (pooled 95–99%), Morigi et al. emphasize the high value of PSMA-PET/CT for the initial staging of high-risk prostate cancer in their non-systematic meta-analysis [26]. This conclusion is very compatible with our finding that PSMA-PET/CT often leads to a change in therapy in high-risk patients. This issue and the high rate of change in initial management are also emphasized by Weiner et al. in a very recent paper [27]. In this publication, the authors also address the high value of PET/CT with regard to treatment planning in relapse cases, with PSMA-PET/CT leading to a change in treatment in many patients, e.g., systemic therapy in 44% of patients instead of local salvage therapy. 

The analysis of the treatments with regard to the different PET/CT findings reveals that in both low- and intermediate-risk patients, the detection of distant metastases, such as bone and organs, only led to an advanced systemic therapy (with ADT/ARPI) in two (2/10; 20%) patients; in seven (7/10; 70%) patients, therapy was continued. It is also interesting to note that in the low-risk patient group, despite the detection of metastases, no treatment was initiated in two patients, most likely as part of a monitoring, wait-and-see approach. This was not found in the intermediate-risk group, where metastases or local findings always led to the initiation or continuation of treatment.

In the intermediate-risk patients with local tumor detection, a locally limited therapy (radiation therapy, local therapy, and surgery) was always chosen; in the case of lymph node metastases, either a locally limited therapy using radiation therapy or, in one presumably advanced case, a systemic therapy with RLT. In the intermediate group, the presentation of bone and organ metastases always had a therapeutic consequence of initiation or continuation of treatment more frequently with systemic therapy (4/7; 57.1%) than local limited therapy (3/7; 42.9%). In this group, therapy was always initiated or continued if metastases or local tumor localization were detected; only patients without tumor detection continued without therapy. This focused analysis of low- and intermediate-risk patients and the revealed results of the scans demonstrate the importance of PET/CT imaging in this setting. Our findings align with the French and EAU-EANM-ESTRO-ESUR-ISUP-SIOG guidelines on prostate cancer, which emphasize the use of PET/CT for staging in intermediate-risk patients, especially when clinical presentation, biochemical recurrence, or recurrence following local treatment indicate an elevated risk [28,29,30]. 

An analysis of the results in our patients with low PSA values (≤2 ng/mL) shows that these patients also frequently had distant metastases (46/153; 30%). It is important to consider the heterogeneity of this collective with a subgroup consisting of patients with increasing PSA levels without or under ADT in the context of the detection of a structural recurrence and also patients under later lines of therapy, which explains the markedly higher rate of distant metastases than in other studies [31]. 

Due to the lack of certainty in differentiating between patients who received the PET/CT for an assessment of a recurrence or an assessment of response to therapy, or restaging for another reason, an additional analysis of these various groups was not conducted.

In the group with a PSA ≤ 0.2 ng/mL, treatment was continued remarkably frequently despite findings in the PET/CT (6/9; 66.7%), most likely due to a presumably low tumor burden, although patients who were PSA-negative or only had low levels despite a high tumor burden must also be considered here, as can be assumed in the case of the patient who was switched from chemotherapy to RLT or continued chemotherapy. In the group with a PSA of 0.21 ≤ 0.5 ng/mL, with local tumor detection and lymph node metastases, a locally limited therapy option in the sense of initiating radiation therapy was used in 5/8 (62.5%) patients, overall, a locally limited therapy option in 7/8 (87.5%) patients, and systemic therapy in only 1/8 (12.5%). In this group, the detection of distant metastases led to locally applicable therapy in 3/7 (42.9%) cases, and systemic therapy was continued in all cases with lymph node and bone metastases. In the patients with a PSA of 0.51 ≤ 1 ng/mL, and distant metastases in 6/12 (50.0%), a locally applicable treatment method was preferred, again most likely due to a low metastatic burden; in 5/12 (41.7%), the treatment was continued. Of the patients with local tumor detection and lymph node metastases, 21/24 (87.5%) were treated with locally applicable therapy. Of the patients with a PSA of 1.01 ≤ 2 ng/mL and distant metastases, only 9/24 (37.5%) received locally applicable treatment methods, considerably fewer patients than in the groups with lower PSA values, which supports the assumption of a lower tumor burden in the other groups in the sense of an oligometastatic situation. Of the patients with local tumor detection and lymph node metastases, 23/37 (62.2%) received locally applicable treatments, considerably fewer patients than in the groups with lower PSA values.

The fact that PSMA-PET/CT will continue to be more important in the future is shown by current studies that investigate and present new clinical indications. In a recent study, Hoffmann et al. also emphasized the real-life added value, particularly with regard to the therapeutic response evaluation of focal therapies and the possible effect on the planning of surgical therapy or radiation therapy [32]. Uslu et al. showed, in the various risk groups, that PSMA-PET/CT can probably also be used in the future with regard to the treatment option of active surveillance, and Shagera et al. recently found that PSMA-PET/CT imaging is valuable for monitoring Xofigo treatment [33,34].

Karpinski et al. were able to show that updated risk groups by PSMA-PET/CT imaging, with predictors such as distant metastases, PSMA expression score, and total lesion count or total tumor volume, can be applied for more accurately predicting the 3- and 5-year overall survival probabilities of prostate cancer than the conventional risk groups [35].

The rise in individualized treatment approaches and the option for ^177^Lutetium-PSMA radionuclide therapy likely contributes to the increased change rates observed in more recent studies as a bias. Additionally, incomplete PSA values and Gleason scores may affect the reliability of our findings, especially given that most patients were of high risk and had PSA levels > 10 ng/mL.

Since we only partly recorded implemented therapies and did not always have the recommendations available, it is unclear whether some patients opted to continue or change their previous therapies, but it may be that in individual cases, patients rejected the recommendation and opted for a different therapy, which was then initiated; this is a possible error that cannot be detected. If ^68^Ga-PSMA-11-PET/CT was indicated solely for metastatic prostate cancer or suspected metastases, the high rate of “no therapy” prior to these diagnostics could reflect an active-surveillance or wait-and-see approach following past treatments. The categorization of cases as “therapy confirmed by ^68^Ga-PSMA-11-PET/CT” might raise some questions, particularly since we could not always ascertain the reasons for or against therapy changes, and other findings might have influenced decisions during the diagnostic period. Additionally, the absence of explicit references to ^68^Ga-PSMA-11-PET/CT does not imply that the examinations lacked value for patients.

Furthermore, the reimbursement of ^68^Ga-PSMA-11-PET/CT is considered a potential source of bias in this study, which cannot be estimated. At the time that the study examinations were conducted in Germany, the examination was often only covered by private health insurance companies or patients on private invoices, so the proportion of patients with an increasing PSA value and thus the more frequent implication of a change in therapy may have predominated.

Due to the design of this study, a statement about a change of progression-free survival in the patients in correlation to the PET/CT is unfortunately not possible but is certainly of interest for the future and the conception of a new study.

## 5. Conclusions

Our study demonstrates the high impact of ^68^Ga-PSMA-11-PET/CT for patients with prostate cancer regarding therapy planning and emphasizes how important the examination is for all patient groups in their individual situation, suggesting that key clinical indicators, including the risk classification, Gleason score, and PSA level, have an impact on treatment decisions. In patients with lower PSA values (≤1 ng/mL), the detection of local tumor and lymph node metastases more often led to locally applicable therapies than in patients with higher PSA values (1.01 ≤ 2 ng/mL). In conclusion, ^68^Ga-PSMA-PET/CT stands as a critical imaging modality that significantly influences treatment decisions, thereby probably enhancing patient outcomes through more targeted and effective therapeutic interventions.

## Figures and Tables

**Table 1:** Basic characteristics.

N = 562		
Year of birth of patients median (min; max)	1947 (1929–1967)	
Activity median (min; max) in MBq	188 (106–343)	
Acquisition time after application in minutes	60 (30–145)	
PSA value in ng/mL	5.1 (0.01–45.839)	
T at initial diagnosis	1	17 (3.0%)
	2	100 (17.8%)
	2a	11 (2.0%)
	2b	12 (2.1%)
	2c	65 (11.6%)
	2x	12 (2.1%)
	3	183 (32.6%)
	3a	43 (7.7%)
	3b	91 (16.2%)
	3c	2 (2.1%)
	3x	47 (8.4%)
	4	17 (3.0%)
	x	245 (43.6%)
N at initial diagnosis	0	193 (34.3%)
	1	83 (14.8%)
	x	286 (50.9%)
M at initial diagnosis	0	63 (11.2%)
	1	21 (3.7%)
	x	478 (85.1%)
D’ Amico risk group	N = 377	
	Low risk	16 (4.2%)
	Intermediate risk	17 (4.5%)
	High risk	344 (91.2%)
Gleason score groups	N = 422	
	Gleason score 5	16 (3.8%)
	Gleason score 6	44 (10.4%)
	Gleason score 7	174 (41.2%)
	Gleason score 8	85 (20.1%)
	Gleason score 9	90 (21.3%)
	Gleason score 10	13 (3.1%)
PSA value groups	N = 482	
	≤0.2	14 (2.9%)
	0.21 ≤ 0.5	27 (5.6%)
	0.51 ≤ 1	45 (9.3%)
	1.01 ≤ 2	67 (13.9%)
	2.01 ≤ 3	26 (5.4%)
	3.01 ≤ 5	61 (12.7%)
	5.01 ≤ 7	37 (7.7%)
	7.01 ≤ 10	37 (7.7%)
	>10	168 (34.9%)

**Table 2:** Adjustments in treatment.

Results N = 562		
Therapy change	Yes	415 (73.8%)
	No	147 (26.2%)
		*In 68, no therapy change explicit because of PET/CT result.*
Therapy before PET/CT	No explicit therapy	227 (40.4%)
	Surgery	13 (2.3%)
	Radiation therapy	40 (7.1%)
	ADT/ARPI	159 (28.2%)
	Chemotherapy	46 (8.2%)
	RLT with 177Lu	59 (10.5%)
	Xofigo	9 (1.6%)
	Seeds	2 (0.4%)
	Local therapies	7 (1.2%)
Therapy after PET/CT	No explicit therapy	56 (10.0%)
	Surgery	32 (5.7%)
	Radiation therapy	157 (27.9%)
	ADT/ARPI	77 (13.7%)
	RLT with 177Lu	117 (20.8%)
	Chemotherapy	57 (10.1%)
	Xofigo	13 (2.3%)
	ARPI (change)	37 (6.6%)
	Local therapies	14 (2.5%)
	RLT with 225Ac	2 (0.4%)

Abbreviations: ADT = androgen deprivation therapy; ARPI = androgen receptor pathway inhibitor, RLT = radioligand therapy, ^177^Lu = ^177^Lutetium, and ^225^Ac = ^225^Actinium.

**Table 3:** Proportion of therapy concepts changed by ^68^Ga-PSMA-11-PET/CT in correlation with the risk groups.

Risk Group	Therapy Change Yes	Therapy Change No	N	Therapy Continued Because of PET/CT	Decision because of PET/CT
Low risk	10	6	16	2	12/16 (75.0%)
Intermediate risk	8	9	17	5	13/17 (76.5%)
High risk	247	97	344	40	287/344 (83.4%)
	272	105	377		

**Table 4:** Treatment adjustments in different Gleason score groups.

Gleason Score	Therapy Change Yes	Therapy Change No	N	Therapy Continued Because of PET/CT	Decision Because of PET/CT
5	11	5	16	1	12/16 (75%)
6	26	18	44	8	34/44 (77.3%)
7	132	42	174	15	147/174 (84.5%)
8	62	23	85	12	74/85 (87.1%)
9	69	21	90	10	79/90 (87.8%)
10	11	2	13	0	11/13 (84.6%)
			422		

**Table 5:** Proportion of therapy concepts changed by ^68^Ga-PSMA-11-PET/CT in correlation to the PSA value.

PSA in ng/mL	Therapy Change Yes	Therapy Change No	N	Therapy Continued Because of PET/CT	Decision Because of PET/CT
≤0.2	5	9	14	2	7/14 (50.0%)
0.21 ≤ 0.5	14	13	27	4	18/27 (66.7%)
0.51 ≤ 1	32	13	45	4	36/45 (80.0%)
1.01 ≤ 2	48	19	67	6	54/67 (80.6%)
2.01 ≤ 3	15	11	26	5	20/26 (76.9%)
3.01 ≤ 5	46	15	61	6	52/61 (85.2%)
5.01 ≤ 7	27	10	37	4	31/37 (83.8%)
7.01 ≤ 10	30	7	37	4	34/37 (91.9%)
>10	140	28	168	18	158/168 (94.0%)
			482		

**Table 6:** Continuation of therapy based on the ^68^Ga-PSMA-11-PET/CT findings.

Therapy Continued Explicitly Due to ^68^Ga-PSMA-11-PET/CT Findings N = 68		
RLT continued N = 37	PSA value in ng/mL	
	Median (Min, Max)	8.7 (0.2–334)
	Gleason Score	
	Median (Min, Max)	8 (6–9)
	Unknown	7 (18.9%)
	6	4 (10.1%)
	7	5 (13.5%)
	8	7 (18.9%)
	9	4 (10.1%)
	D’Amico	
	Unknown	15 (40.5%)
	Intermediate risk	3 (8.1%)
	High risk	19 (51.4%)
	PET/CT result	
	LN Metastases	5 (13.5%)
	Bone Metastases	10 (27.0%)
	LN + Bone Metastases	21 (56.8%)
	Organ Metastases	5 (13.5%)
No therapy continued N = 12	PSA value in ng/mL	
	Median (Min, Max)	1.7 (0.24–13.5)
	Gleason Score	
	Median (Min, Max)	7 (5–9)
	Unknown	2 (16.7%)
	5	1 (8.3%)
	6	2 (16.7%)
	7	6 (50.0%)
	9	1 (8.3%)
	D’Amico	
	Unknown	2 (16.7%)
	Low risk	2 (16.7%)
	Intermediate risk	1 (8.3%)
	High risk	7 (58.3%)
	PET/CT result	
	No evidence of disease	9 (75.0%)
	LN Metastases	2 (16.7%)
	Local tumor	1 (8.3%)
ADT/ARPI continued N = 6	PSA value in ng/mL	
	Median (Min, Max)	2.55 (0.3–546)
	Gleason Score	
	Median (Min, Max)	8 (7–9)
	Unknown	1 (16.7%)
	7	2 (33.3%)
	8	1 (16.7%)
	9	2 (33.3%)
	D’Amico	
	Unknown	1 (16.7%)
	High risk	5 (83.3%)
	PET/CT result	
	No evidence of disease	1 (16.7%)
	LN Metastases	4 (66.7%)
	LN + Bone Metastases	1 (16.7%)
	Local tumor	1 (16.7%)
Radiation therapy continued N = 5	PSA value in ng/mL	
	Median (Min, Max)	8 (0.78–26.3)
	Gleason Score	
	Median (Min, Max)	7 (6–9)
	Unknown	2 (40%)
	6	1 (20%)
	7	1 (20%)
	9	1 (20%)
	D’Amico	
	Unknown	1 (20%)
	Intermediate risk	1 (20%)
	High risk	3 (60%)
	PET/CT result	
	No evidence of disease	1 (20%)
	LN Metastases	2 (40%)
	LN + Bone Metastases	1 (20%)
	Local tumor	1 (20%)
Chemotherapy continued N = 5	PSA value in ng/mL	
	Median (Min, Max)	13.2 (0.04–88)
	Gleason Score	
	Median (Min, Max)	8 (6–9)
	6	1 (20%)
	7	1 (20%)
	8	2
	9	1 (20%)
	D’Amico	
	Unknown	1 (20%)
	High risk	4 (80%)
	PET/CT result	
	LN Metastases	1 (20%)
	Bone Metastases	2 (40%)
	LN + Bone Metastases	1 (20%)
	Organ Metastases	2 4(0%)
Local therapy continued N = 2	PSA value in ng/mL	
	Median (Min, Max)	0.8 (0.5–1.1)
	Gleason Score	
	Median (Min, Max)	8 (8.8)
	8	2 (100%)
	D’Amico	
	High risk	2 (100%)
	Bone Metastases	2 (100%)
Xofigo continued N = 1	PSA value in ng/mL	25
	Gleason Score	
	9	1 (100%)
	D’Amico	
	High risk	1 (100%)
	Bone Metastases	1 (100%)

Abbreviations: LN = lymph node.

**Table 7:** Therapy decision groups after ^68^Ga-PSMA-11-PET/CT.

Therapy Decision N = 562		
Therapy continued due to PET/CT N = 68	PSA value in ng/mL	
	Median (Min, Max)	5 (0.04–3349)
	Gleason Score	
	Median (Min, Max)	7 (5–9)
	Unknown	22 (32.4%)
	5	1 (1.5%)
	6	8 (11.8%)
	7	15 (10.3%)
	8	12 (17.6%)
	9	10 (14.7%)
	10	0 (0%)
	D’Amico	
	Unknown	20 (29.4%)
	Low risk	2 (2.9%)
	Intermediate risk	5 (7.4%)
	High risk	41 (60.3%)
	No evidence of disease	11 (16.2%)
	Lymph node metastases	13 (19.1%)
	Bone metastases	15 (22.1%)
	Lymph node + bone metastases	25 (36.8%)
	Local tumor (prostate gland/prostate bed)	3 (4.4%)
	Organ metastases	7 (10.3%)
Therapy modified N = 415	PSA value in ng/mL	
	Median (Min, Max)	6 (0.01–14000)
	Gleason Score	
	Median (Min, Max)	7 (5–10)
	Unknown	96 (23.1%)
	5	11 (2.7%)
	6	28 (6.7%)
	7	135 (32.5%)
	8	66 (15.9%)
	9	69 (16.6%)
	10	10 (2.4%)
	D’Amico	
	Unknown	142 (34.1%)
	Low risk	11 (2.7%)
	Intermediate risk	9 (2.2%)
	High risk	253 (61.0%)
	No evidence of disease	18 (4.3%)
	Lymph node metastases	117 (28.2%)
	Bone metastases	85 (20.5%)
	Lymph node + bone metastases	127 (30.6%)
	Local tumor (prostate gland/prostate bed)	80 (19.2%)
	Organ metastases	20 (4.8%)
Therapy continued without explicit reference N = 79	PSA value in ng/mL	
	Median (Min, Max)	2 (0.01–452)
	Gleason Score	
	Median (Min, Max)	7 (5–10)
	Unknown	22 (29.3%)
	5	4 (5.1%)
	6	8 (10.1%)
	7	24 (30.4%)
	8	7 (8.9%)
	9	11 (13.9%)
	10	3 (3.8%)
	D’Amico	
	Unknown	23 (29.1%)
	Low risk	3 (3.8%)
	Intermediate risk	3 (3.8%)
	High risk	50 (63.3%)
	No evidence of disease	30 (38.0%)
	Lymph node metastases	17 (21.5%)
	Bone metastases	18 (22.8%)
	Lymph node + bone metastases	7 (8.9%)
	Local tumor (prostate gland/prostate bed)	6 (1.4%)
	Organ metastases	7 (8.9%)

**Table 8:** Therapies based on the ^68^Ga-PSMA-PET/CT findings in low-risk patients.

PET/CT Result	Therapy
Bone and organ metastases	1 ADT/ARPIs continued
Bone metastases	1 radiation therapy continued
Local tumor detection	4 no therapy to radiation therapy 1 no therapy continued 1 seeds to surgery
Lymph node and bone metastases	1 chemotherapy to RLT 1 seeds to ADT/ARPIs
Lymph node metastases	3 no therapy continued 1 no therapy to ADT/ARPIs 1 chemotherapy to radiation therapy
Lymph node, bone, and organ metastases	1 ADT/ARPIs to chemotherapy

**Table 9:** Therapies based on the ^68^Ga-PSMA-PET/CT findings in intermediate-risk patients.

PET/CT Result	Therapy
Bone metastases	1 no therapy to chemotherapy 3 RLT continued 1 no therapy to radiation therapy
Local tumor detection	2 no therapy to radiation therapy
Lymph node and bone metastases	1 no therapy to ADT/ARPIs 1 radiation therapy to PRRT
Lymph node metastases	1 surgery to radiation therapy 1 radiation therapy continued 1 ADT/ARPIs continued
Lymph node metastases and local tumor detection	1 no therapy to radiation therapy
No tumor lesion is imageable	3 no therapy continued 1 radiation therapy continued

**Table 10:** Therapies based on the ^68^Ga-PSMA-PET/CT findings in patients with a PSA ≤ 0.2 ng/mL.

PET/CT Result	Therapy
Bone metastases	1 Radiation Therapy to ARPI 1 ADT/ARPIs continued 1 no therapy continued
Local tumor detection	1 ADT/ARPIs continued
Lymph node metastases	1 no therapy continued 1 RLT continued 1 chemotherapy continued 1 chemotherapy to RLT 1 RLT to no therapy
No tumor lesion imageable	1 RLT to no therapy 3 no therapy continued 1 ADT/ARPIs continued

**Table 11:** Therapies based on the ^68^Ga-PSMA-PET/CT findings in patients with a PSA of 0.21 ≤ 0.5 ng/mL.

PET/CT Result	Therapy
Bone metastases	1 local therapy continued 1 no therapy to radiation therapy
Local tumor detection	1 surgery continued 1 ADT/ARPIs to local therapy 1 no therapy to radiation therapy 1 surgery to radiation therapy
Lymph node and bone metastases	1 RLT continued 1 chemotherapy continued 1 ADT/ARPIs continued
Lymph node metastases	3 no therapy to radiation therapy 1 surgery to RLT
No tumor lesion imageable	7 no therapy continued 2 surgery to radiation therapy 1 ADT/ARPIs continued 1 ADT/ARPIs to radiation therapy 1 no therapy to radiation therapy
Organ metastases	1 local therapy continued 1 radiation therapy to ADT/ARPIs

**Table 12:** Therapies based on the ^68^Ga-PSMA-PET/CT findings in patients with a PSA of 0.51 ≤ 1 ng/mL.

PET/CT Result	Therapy
Bone metastases	1 no therapy to ADT/ARPIs 2 RLT continued 1 chemotherapy continued 1 ADT/ARPIs to chemotherapy 1 local therapy continued 1 surgery to radiation therapy 1 no therapy to radiation therapy
Local tumor detection	2 ADT/ARPIs to radiation therapy 4 no therapy to radiation therapy
Lymph node and bone metastases	1 radiation therapy continued 1 ADT/ARPIs to radiation therapy 1 no therapy to ADT/ARPIs
Lymph node metastases	13 no therapy to radiation therapy 2 no therapy to surgery 1 ADT/ARPIs to RLT 2 no therapy continued
No tumor lesion imageable	6 no therapy continued 1 ADT/ARPIs to surgery 2 no therapy to radiation therapy
Organ metastases	1 no therapy to radiation therapy

**Table 13:** Therapies based on the 68Ga-PSMA-PET/CT findings in patients with a PSA of 1.01 ≤ 2 ng/mL.

PET/CT Result	Therapy
Bone metastases	3 no therapy to radiation therapy 5 no therapy to ARPIs 3 radiation therapy to ADT/ARPIs 2 RLT continued 1 local therapy continued 1 local therapy to radiation therapy 2 no therapy continued
Local tumor detection	1 no therapy to surgery 2 no therapy to local therapy 1 radiation therapy to surgery 2 no therapy to radiation therapy 1 ADT/ARPI to radiation therapy
Local tumor detection and organ metastases	1 no therapy to radiation therapy
Lymph node and bone metastases	1 local therapy continued 1 no therapy to radiation therapy 1 ADT/ARPIs to radiation therapy 1 no therapy to ADT/ARPIs
Lymph node and bone metastases and local tumor detection	1 radiation therapy to ADT/ARPIs
Lymph node metastases	1 no therapy to ADT/ARPIs 7 ADT/ARPIs to radiation therapy 3 ADT/ARPIs continued 1 ADT/ARPIs to ARPIs (change) 2 ADT/ARPIs to RLT 3 ADT/ARPIs to chemotherapy 2 chemotherapy to RLT 3 no therapy to local therapy 1 radiation therapy continued 1 no therapy continued 1 no therapy to surgery 1 no therapy to radiation therapy
Lymph node metastases and local tumor detection	2 no therapy to radiation therapy 1 no therapy to local therapy 1 ADT/ARPIs continued
Lymph node, bone, and organ metastases	1 ADT/ARPIs to RLT
No tumor lesion imageable	1 no therapy continued 1 radiation therapy continued 1 ADT/ARPIs continued 1 no therapy to ADT/ARPIs 2 no therapy to radiation therapy

## Data Availability

The data that support the findings of this study are available upon reasonable request (F.E.).
